# Can Deep Learning Blind Docking Methods be Used to Predict Allosteric Compounds?

**DOI:** 10.1021/acs.jcim.5c00331

**Published:** 2025-04-01

**Authors:** Eric A. Chen, Yingkai Zhang

**Affiliations:** † Department of Chemistry, New York University, New York, New York 10003, United States; ‡ Simons Center for Computational Physical Chemistry at New York University, New York, New York 10003, United States; § NYU-ECNU Center for Computational Chemistry, Shanghai 200062, China

## Abstract

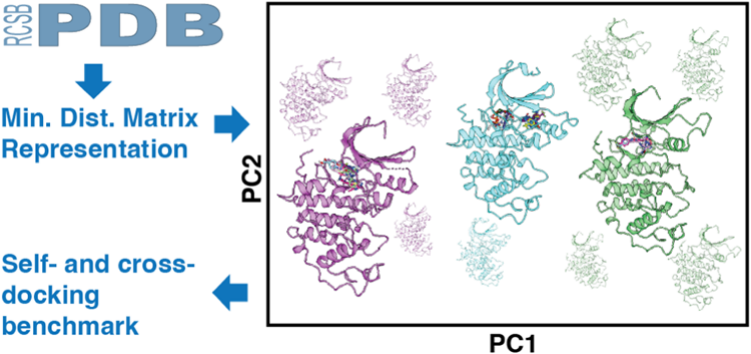

Allosteric compounds offer an alternative mode of inhibition to orthosteric compounds with opportunities for selectivity and noncompetition. Structure-based drug design (SBDD) of allosteric compounds introduces complications compared to their orthosteric counterparts; multiple binding sites of interest are considered, and often allosteric binding is only observed in particular protein conformations. Blind docking methods show potential in virtual screening allosteric ligands, and deep learning methods, such as DiffDock, achieve state-of-the-art performance on protein–ligand complex prediction benchmarks compared to traditional docking methods such as Vina and Lin_F9. To this aim, we explore the utility of a data-driven platform called the minimum distance matrix representation (MDMR) to retrospectively predict recently discovered allosteric inhibitors complexed with Cyclin-Dependent Kinase (CDK) 2. In contrast to other protein complex representations, it uses the minimum residue–residue (or residue–ligand) distance as a feature that prioritizes the formation of interactions. Analysis of this representation highlights the variety of protein conformations and ligand binding modes, and we identify an intermediate protein conformation that other heuristic-based kinase conformation classification methods do not distinguish. Next, we design self- and cross-docking benchmarks to assess whether docking methods can predict both orthosteric and allosteric binding modes and if prospective success is conditional on the selection of the protein receptor conformation, respectively. We find that a combined method, DiffDock followed by Lin_F9 Local Re-Docking (DiffDock + LRD), can predict both orthosteric and allosteric binding modes, and the intermediate conformation must be selected to predict the allosteric pose. In summary, this work highlights the value of a data-driven method to explore protein conformations and ligand binding modes and outlines the challenges of SBDD of allosteric compounds.

## Introduction

Allostery is a perturbation, such as ligand binding, distal to the active site that alters the conformational ensemble to modulate the protein function.^1,2^ This allosteric phenomenon has motivated the development of therapeutics that are noncompetitive with the native ligand, avoid orthosteric resistance, improve selectivity, and limit off-target toxicity.^3^ Allosteric inhibitors shift the active/inactive enzymatic equilibrium to the inactive state, thus disabling protein function. This is in contrast to orthosteric inhibitors, whose mechanism of inhibition is to outcompete the native ligand. Recently, there has been considerable interest and promise in the development of allosteric kinase inhibitors.^4^

Deep learning protein–ligand binding pose prediction methods enable the structure-based drug design (SBDD) of allosteric binders.^5^ In this work, we focus on blind docking methods, where the ligand pose search space is unrestricted. Traditional docking methods like Vina and Lin_F9 rely on crafted scoring functions that rate binding poses and optimization algorithms that search for the global maximum of the scoring function.^6−8^ Deep learning blind docking methods are principally different in that they rely on trained parameters to build the scoring function. We focus on diffusion-based generative methods, which are likelihood-based models that learn a data distribution by learning the reverse process of the forward diffusion process.^9,10^ These methods vary depending on how they treat the receptor. Rigid-receptor docking methods such as DiffDock (2023)/-S/-L treat the protein statically.^11,12^ DynamicBind, a flexible pocket docking method, jointly predicts protein side-chain and secondary structures movement and ligand binding pose.^13^ These deep learning methods have garnered significant interest and have achieved state-of-the-art performance on benchmarks; therefore, we evaluate their ability to predict allosteric binders.

Improvements in the accuracy of blind docking methods assist in scenarios in which the target binding site is unknown. In drug discovery campaigns, where the binding site is chosen or determined by pocket-searching techniques, the alternative approach to blind docking is known-pocket docking. In this approach, the search space is restricted to the specific binding pocket and can have a strong performance.^14−16^ These diffusion generative models have also been adapted for known-pocket docking.^17^ Even if the binding site is known, a blind docking method could still improve the hit enrichment of allosteric compounds compared to known-pocket docking. If a blind docking method can robustly discriminate between orthosteric and allosteric compounds, one can filter away the compounds more likely to be orthosteric binders, resulting in a set of compounds more likely to be allosteric.

Although deep learning models can generate protein–ligand poses, the selection of the receptor structure is nontrivial because proteins are dynamic and show conformational diversity. For example, kinases are known to fold into one active conformation but multiple inactive conformations. These conformations regulate cellular physiology and are key factors for protein–protein and protein–ligand interactions. There are a few heuristic-based kinase conformation classification methods.^18−21^ While these methods can classify structures into multiple categories, they lack applicability to other proteins and require *a priori* knowledge of the structural determinants of kinase activation/inactivation. These classification methods highlight the DFG motif and the αC-helix region. This limits the capability of the classification method to process features outside the catalytic binding domain and classify protein structures based on diverse allosteric sites.

**Figure 1:**
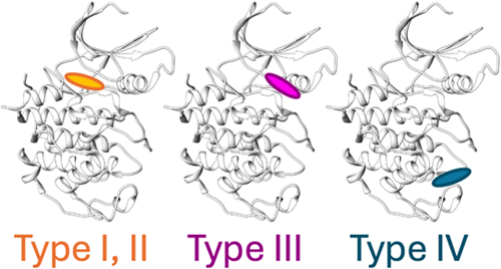
Kinase binding modes and their respective binding pockets. Type I and II ligands bind in the ATP-binding site. Type III ligands bind in the site adjacent to the ATP site. Type IV ligands are a more general category of allosteric binders where binding occurs at any site proximal to the orthosteric site.

Kinase inhibitor binding modes are also well-categorized. Type I and II inhibitors are orthosteric binders to the active and inactive conformations, respectively. Type III and IV inhibitors are allosteric binders at the site adjacent to or proximal to the ATP site, respectively (Figure 1).^22^ Some methods use distance-based heuristics or rely on superimposition to define the kinase binding modes.^14,15,21^ Other methods are principally ligand-based methods and do not take a structural approach.^23−25^

We introduce the minimum distance matrix representation (MDMR) platform to establish the challenges of structure-based blind docking of allosteric compounds with thorough benchmarking (Figure S1). A receptor conformation is represented as a matrix of pairwise residue–residue distances. The distance, when defined as the minimum distance, is a proxy for inter-residue interactions. Analysis of this data representation identifies the distinguishing interactions among the receptor conformations. This can also extend to distinguishing ligand binding locations when measuring the minimum residue–ligand distances. The combined analysis of the receptor conformations and ligand binding locations then guides the development of two benchmarks that assess the capability of blind docking methods to accurately predict binding poses. The self-docking benchmark partitions docking success into orthosteric and allosteric binding modes. The cross-docking benchmark mimics a prospective study by relying on a receptor time-split and evaluates docking success by permutations of the ligand binding mode and receptor conformation. The results from this study can direct future examinations of other protein systems, suggest areas of improvement for blind docking methods, or provide insight into planning prospective tests.

The focus of our study is Cyclin-Dependent Kinase (CDK) 2, a promising therapeutic target that is associated with the proliferation of cancer cells and is also identified as a male nonhormonal contraceptive target.^26−29^ Notably, CDK2 relies on interaction with cyclin proteins for activation. The diverse set of publicly available CDK2 structural data has led to the development of SBDD strategies that evaluate the role of receptor conformations, such as ensemble docking^30−32^ and cross-docking.^33−37^ Much of the effort thus far focuses on the highly conserved ATP-binding site, but there has yet to be an evaluation of computational approaches to the discovery and prediction of allosteric inhibitors to CDK2 (Figure S2).^38,39^ Researchers are interested in the allosteric inhibition for CDK2 because it can lead to improved selectivity compared to orthosteric inhibitors.^29,40^ We retrospectively apply our platform to the discovery of Type III inhibitors bound to CDK2 that were resolved by Faber et al.^41,42^

## Methods

### Selection of Matrix Embedding

The MDMR allows us to classify and assess the conformational diversity of the structural ensemble. The first stage is to obtain and process the structural entries from the Protein Data Bank of CDK2.^43^ For detailed information, see the supporting methods. Because we only focus on one target, we take the entire length of the CDK2 UniProt sequence, amounting to 298 residues. We then embed each receptor conformation into an *n* × *n* pairwise residue–residue distance matrix, where *n* is the number of residues and the distance is the minimum residue–residue distance. We primarily explore the utility of the minimum distance matrix representation because of its natural extension to include ligands. For the ligand binding location embedding of each ligand, we calculated the receptor–ligand minimum distance vector of length *n*. The data matrices and vectors are cleaned by simply removing the residues from the data structures that are not available in every structure.

### Dimension Reduction and Clustering

We apply two unsupervised machine learning methods from scikit-learn 1.3.0, Principal Component Analysis (PCA) and hierarchical density-based spatial clustering of applications with noise (HDBSCAN), to the receptor-only distance matrices or the ligand-only distance vectors.^44−47^ The combination of PCA and density-based clustering methods has been successfully applied to molecular dynamics (MD) sets in the past.^48,49^

PCA reduces the dimensionality of the data while maximizing the variance. The algorithm evaluates principal components (PC) and uncorrelated linear functions of the data set along which variance is maximized. The first PC is associated with the most variance along this direction, and subsequent PCs are orthogonal to the first one with descending variance. We retain the largest PCs that cumulatively contribute to at least 0.75 of the explained variance ratio. While the loss of information is natural when discarding the remaining PCs, we anticipate that the retained PCs are sufficient to highlight the variability of receptor conformations or ligand binding locations in the matrices or vectors, respectively. The data representations are projected onto PCs, and the structural similarities and dissimilarities are observed. By projecting each representation onto a few PCs rather than projecting onto the *n*(*n* – 1)/2 unique features of the matrix, the complexity and dimensionality of the representation are greatly reduced.

The PCA-projected data points are then clustered using HDBSCAN, an algorithm that is particularly useful for clusters of variable density levels. It is built off of density-based spatial clustering of applications with noise (DBSCAN) and relies on the observation that cluster centroids exhibit a higher local density compared to their neighbors and are far from other points of higher density. The algorithm creates a *mutual reachability graph* where any point *X*_*i*_ is a node in the graph, and the edges are the *mutual reachability distances*, *d*_mreach_ (1). This is defined as
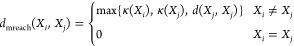
1where a *core-distance* function κ(*X*_*i*_) is defined as the max Euclidean distance between *X*_*i*_ and its *k*th nearest neighbor samples and Euclidean distance *d*(·, ·). In practice, this reshapes the space to make the sparse points more sparse without altering the distance between dense points. This graph is then transformed into a dendrogram by constructing a minimum spanning tree whose edges are sorted by *d*_mreach_. At this stage, DBSCAN diverges from HDBSCAN, where a selected global threshold, ϵ, is selected to cut the dendrogram and define the clusters.^50,51^

In HDBSCAN, clusters are instead defined with the *excess of mass* method, which groups nodes into clusters if they stay persistently dense despite the dendrogram splitting.^45,52^ This creates a metric for each cluster *C*_*i*_ called cluster *stability S*(*C*_*i*_), which is defined as
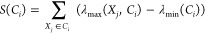
2where λ = 1/d. The method traverses the dendrogram bottom up, treating each node as a possible cluster and summing the stability values until the clusters with the highest stabilities on each branch are found and selected. To frame this method in context to DBSCAN, this creates a hierarchy for all possible ϵ thresholds with respect to *S*(*C*_*i*_), thus resulting in clusters of different density levels.

In our case, we set the primary parameter of HDBSCAN, *k* (minimum_samples), to the default value of 5 and include an optional threshold parameter ϵ̂ = cluster_selection_epsilon = 10.0, which prevents clusters below the given threshold from splitting up any further.^52^ We set this ϵ̂ value because we observe spatially close microclusters when we want one or two clusters to represent each conformation.

### Normalized Standardized Mean Difference

Following dimension reduction of the receptor-only distance matrices and clustering, we perform further analysis to highlight interactions as the defining characteristic that elucidates similarities and dissimilarities between receptor conformations. With the structures classified into multiple clusters, the distance pairs that differentiated the two clusters can be determined using a standardized mean difference (SMD) metric (3). This SMD metric quantifies the effect size of each *i*th and *j*th residue–residue distance pair between clusters *x* and *y*.
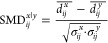
3
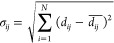
4where  is the average distance between *i*th and *j*th residues of cluster *x*, and σ_*ij*_^*x*^ is the standard deviation of the *ij* distance pairs from the *N* structures in cluster *y*. To further bias the residue–residue distances that form interactions, the SMD metric is normalized by dividing by the difference between the minimum *ij* distance pair and normalization value α.
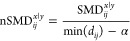
5This α value is set to 1.5 to reflect the possibility of hydrogen atoms that are often not observed in X-ray crystal structures, or it can be set to 0 when analyzing NMR structures or structures with hydrogen atoms present. A high nSMD value reflects a difference in the distance pair between clusters.

### Conformation-Based Loop Modeling

Prior to performing blind docking, we model missing residue segments with a template-based approach with Modeller that selects a similar protein conformation as the template.^53,54^ This approach is herein termed *conformation-based loop modeling*. The selection of template structures takes advantage of the utility of the MDMR to robustly cluster and classify protein structures by their respective conformations. This method is useful for repairing structures that have similar conformations from the same or related protein.

The selection of template structures begins following the dimension reduction and clustering of receptor-only structures. First, the missing residues for each PDB structure are collected. Any unresolved terminal residues are excluded. Then, each structure is assigned to template structures based on the closest PCA-projected data points, the resolution of corresponding structures, and cluster identity.

The reconstruction of the missing loop regions uses an adapted version of the AutoModel class in Modeller. A model with the corrected sequence is quickly rebuilt. Any noncanonical amino acids are converted back to the original amino acid as defined by the UniProt sequence. The missing regions are reconstructed based on the assigned template structures. Five models are generated with hydrogens and then optimized with the built-in protocols using the variable target function method^55^ and molecular dynamics (MD) refinement with simulated annealing.^54,56^ The best model with the lowest DOPE score is selected for docking.

### Blind Docking

For the traditional docking methods Vina and Lin_F9, each receptor conformation is prepared using pdb2pqr v3.5.2 to determine protonation states, add hydrogens, and rebuild any missing side chains.^57^ Next, MGLTools 1.5.6 prepare_receptor4.py script is used to add Gasteiger charges and AutoDock Vina types and remove the nonpolar hydrogens.^58^ The ligand preparation procedure begins with obtaining the SMILES representation of each compound. RDKit version 2020.03.1 is used to add hydrogens, generate initial three-dimensional (3D) conformers using ETKDG, and then optimize with the MMFF94 force field.^59,60^ To reduce the redundancy of each set of conformers for a given ligand, a matrix is created by pairwise aligning each conformer and calculating root-mean-squared-deviation (RMSD). The matrix is then clustered using the Butina algorithm using a 2 Å threshold.^61^ Clustering strategies that use single-reference alignment and avoid pairwise RMSD calculations may also be used here to improve computational scalability for large and flexible molecules.^62^ Then, each ligand conformer is prepared by using Meeko 0.3.3 to add Gasteiger charges and AutoDock Vina types and remove the nonpolar hydrogens.^63^

The ligand conformers are then docked using the traditional docking methods with the hyperparameters listed below.

- Vina 1.2.3:^6,7^ Blind docking is performed using exhaustiveness = 64 and num_modes = 9. The search box is generated around the entire receptor following the autobox procedure outlined in Smina using a + 4 Å buffer. The poses from each conformer are consolidated and ranked with the Vina scoring function for docking evaluation.
- Lin_F9:^8^ Blind docking is performed using the Smina docking method with the Lin_F9 scoring function using exhaustiveness = 64 and num_modes = 9. The search box is generated around the receptor with a + 4 Å buffer. The poses from each conformer are consolidated and ranked with the Lin_F9 scoring function for docking evaluation.

For the deep learning blind docking methods, the ligand is docked onto the receptor starting from the SMILES representation with the hyperparameters listed below.

- DiffDock (2023):^11^ 10 samples per complex are generated using default parameters and ranked with the provided confidence model.
- DiffDock-L:^12^ 10 samples per complex are generated using default parameters and ranked with the provided confidence model.
- DiffDock-S:^12^ 10 samples per complex are generated using default parameters and ranked with the provided confidence model.
- DynamicBind:^13^ 10 samples per complex are generated using default parameters and ranked with the provided affinity prediction model.

For the combination of DiffDock and Lin_F9 Local Re-Docking (LRD), the preparation begins by following the preparation for the deep learning blind docking methods. Next, receptor and ligand preparation for traditional docking is performed. The hyperparameters for this strategy are listed below.

- DiffDock (2023)^11^ + LRD:^8^ First run DiffDock with default parameters, then perform Lin_F9 LRD. Lin_F9 LRD performs traditional docking using the Lin_F9 scoring function with the box that is autogenerated on the DiffDock pose with +4 Å buffer added using exhaustiveness = 8 and num_modes = 1. The poses are ranked with the Lin_F9 scoring function.

We treat the receptor rigidly for all blind docking methods presented here except for DynamicBind.^13^

## Results and Discussion

### Minimum Distance Matrix Approach to Embed Protein Conformation

Key to the SBDD of allosteric inhibitors is an exploration of the conformational states of kinases. The MDMR allows us to cluster and assess the conformational diversity of the structural ensemble. 453 available X-ray crystallography and Cryo-EM experimental entries before 04/13/2023 containing at least one structure of CDK2 (UniProt ID: P24941) are downloaded from the RCSB database.^43,64^ Parsing only the CDK2 receptor structure from each file amounts to 542 structures of CDK2. Each CDK2 structure is represented as a pairwise residue–residue minimum distance matrix.

**Figure 2:**
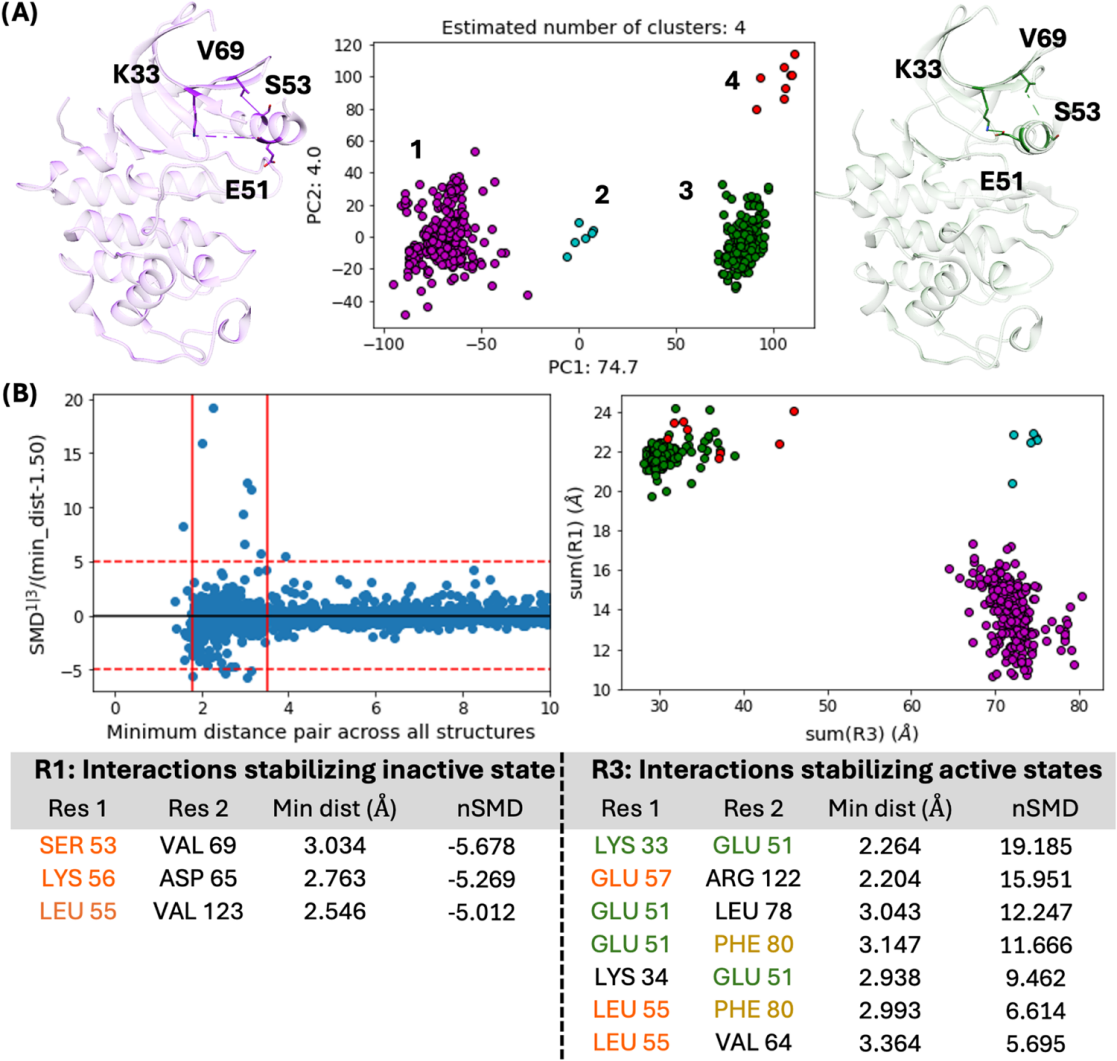
(A) Receptor-Only Principal Component Analysis (PCA) and Hierarchical Density-Based Spatial Clustering of Applications with Noise (HDBSCAN) clustering. Each available CDK2 protein conformation is represented by a pairwise residue–residue minimum distance matrix and then input into PCA and HDBSCAN. Example structures from the inactive cluster (c1^R^; purple) and the active cluster (c3^R^; green) with the most distinguishing distance pair shown in solid/dashed lines between residues shown in sticks. (B) *Upper left:* Selected residue–residue distance pairs that have 1.8 Å < min(*d*_*ij*_^1|3^) < 3.5 Å and nSMD^1|3^ > 5 (R3) or nSMD^1|3^ < 5 (R1) reflect interactions that distinguish cluster 1 from 3. The solid and dashed red lines on the plot indicate the thresholds for selecting the R1 and R3 interactions of the minimum distance and nSMD, respectively. *Upper right:* These distances were summed to separate the CDK2 structures along two axes, R1 and R3. *Below:* Table of selected distance pairs and the minimum distance and nSMD of the corresponding cluster. The color of the text corresponds to the region of the protein, as depicted in Figure S2.

Next, PCA and HDBSCAN are used to analyze and observe the receptor conformational space. This procedure classifies similar conformations together and distinguishes dissimilar conformations by virtue of their interactions. The resulting analysis of the CDK2 receptor-only data set suggests that there are 4 main clusters with clear separation across PC1 (Figure 2A). The binding partner details of each cluster indicate that the green (c3^R^) and red (c4^R^) clusters are comprised of structures complexed with cyclin and SPY1 activator proteins (Figure S3). Although these clusters are distinguished along PC2, the explained variance ratio of PC2 is low, suggesting the variance of the features along this principal component is not as significant as the variance along PC1. Thus, these clusters are grouped together and labeled the active state. The purple (c1^R^) cluster is comprised of structures often bound by inhibitors, and the cyan (c2^R^) cluster is comprised of structures bound by the allosteric probe, 8-anilino-1-naphthalenesulfonate (ANS; PDB ligand ID: 2AN). These are labeled as the inactive and intermediate states, respectively. The separate clustering of structures bound by the ANS reflects the observations by Betzi et al. that the ANS ligand induces or binds to a different conformation that is not observed in cyclin-bound states or typical inactive states.^65^ We note that none of the aforementioned kinase conformation classification methods distinguish these intermediate structures (Tables S1–S3).^18−21^ In summary, the receptor-only clustering reveals 4 clusters where c3^R^ and c4^R^ are denoted as the active state, c1^R^ as the inactive state, and c2^R^ as the intermediate state.

This minimum distance matrix is one of many possible representations of a protein conformation to perform conformational ensemble analysis. One related approach is Essential Mode Analysis (EMA), which uses Cα atomic fluctuations about an averaged structure as the input to the PCA.^66−68^ EMA + HDBSCAN distinguishes the active and inactive states across PC1 (Figure S4A). Comparing our MDMR clustering with the EMA plot reveals that EMA is unable to distinguish the c2^R^ intermediate state (Figure S4B). Other methods first require superimposition or RMSD,^69−71^ use differential geometry,^39,72−74^ or take combinations of input features.^75,76^ Other matrix approaches, such as contact maps or Cα–Cα distance matrices, or other residue selections can also be informative matrix representations (Figure S5). The advantage of the minimum residue–residue distance structural representation presented here is that it is both superposition independent and incorporates the combined effect of backbone and side chain changes.

Next, a normalized standardized mean difference metric (nSMD) for each distance pair highlights the variance in the matrices and identifies the significant interactions distinguishing the conformation clusters. The nSMD between the active (c3^R^: green) and an inactive cluster (c1^R^: purple) is calculated and plotted against the minimum distance of that distance pair across all of the structures (Figure 2B). All of the distance pairs with a nSMD > 5 and 1.8 Å < minimum distance < 3.5 Å are categorized as R3, interactions that occur in structures in the active cluster (c3^R^) but not the inactive cluster (c1^R^) (Figure 2B). The highlighted interactions reflect important structural and catalytic roles. The top-ranking nSMD interaction is the important LYS33–GLU51 salt bridge, which is a prerequisite for the formation of active conformation in protein kinases and is the defining feature of the αC helix-in (αC-in) conformation.^27^ This interaction is not observed in the inactive cluster, and the GLU51 side chain is turned away from the active site, consistent with the αC helix-out (αC-out) conformation. Other notable interactions include the electrostatic interaction between GLU57 and ARG122 and regulatory spine residue LEU55 (RS3) hydrophobic interactions with the shell residues VAL64 (Sh1) and PHE80 (Sh2) as defined by Kornev et al.^27,77^ Conversely, distance pairs with a nSMD < −5 and 1.8 Å < minimum distance < 3.5 Å are categorized as R1, interactions that occur in the structures in the inactive cluster but not the active cluster (Figure 2B). These R1 distances mostly reflect interactions between residues in the αC-helix and β5 sheet. These interactions appear to stabilize the αC-out conformation and force GLU51 to be solvent-exposed. The histograms of the distance distributions also confirm these observations (Figure S6). This analysis underscores the interactions that distinguish or qualify these clusters of structures and the common αC-in vs αC-out nomenclature, which is necessary for distinguishing the active CDK2 conformation from the inactive conformation.^27^

The sum of the R3 distances versus the sum of the R1 distances plot is an informative plot about the interactions (Figure 2B). Here, the active state structures are all clustered distinctly from the inactive state structures clustered. The ANS-bound, intermediate state structures cluster to the top right of the plot, indicating that both R1 and R3 distances are broken and highlighting the uniqueness of the conformation. In the crystal structure of an intermediate conformation, the CDK2–ANS ternary complex (PDB ID: 3PXF), two ANS ligands bind to the allosteric pocket adjacent to the ATP site between αC-helix and the β4 and β5 sheets. The primary ANS ligand interrupts important R1 and R3 distances by interacting with LYS33, PHE80, LYS56, and VAL64, while the secondary ANS ligand interrupts R3 interactions by interacting with LYS56 and VAL69 (Figure S7).

We perform a triplicate of 500 ns MD simulations initialized from the active, intermediate, and inactive conformations. For detailed information on the simulations and analysis, refer to the supporting methods. We produce density histograms of the sum(R1) vs sum(R3) to track the conformational state of CDK2 during the simulations (Figure S8). The results indicate that all three active, intermediate, and inactive conformations are metastable in the *holo* conformation because they remain in a state similar to their respective crystal structures (Figure S8A–C). *Apo* simulations starting from the intermediate conformation populate the inactive regime. This suggests that the intermediate conformation may not be observed natively, and the ANS probe is required to be bound for the conformation to retain the metastable state where both the R1/R3 distances are broken (Figure S8C,D).

The MDMR has been applied to other systems as well. It has been used to distinguish the active and autoinhibited conformations of the FGFR kinase isoforms and elucidate how mutations of the gatekeeper residue alter the conformations defining interactions.^78^ It has also assisted in the identification of the binding site of a hit compound to the SARS-CoV-2 nsp13 by classifying the receptor conformation and performing blind ensemble docking.^79^ In both of these cases, this approach details downstream analyses in the context of the conformational ensemble. Without additional modifications, MDMR may not be able to identify conformations in contexts where transient side chain interactions dominate. For example, loop regions within molecular dynamics simulations can introduce a lot of noise and deter meaningful clustering.

### Self- and Cross-Docking Benchmarking Strategy to Assess Deep Learning Blind Docking Methods

**Figure 3:**
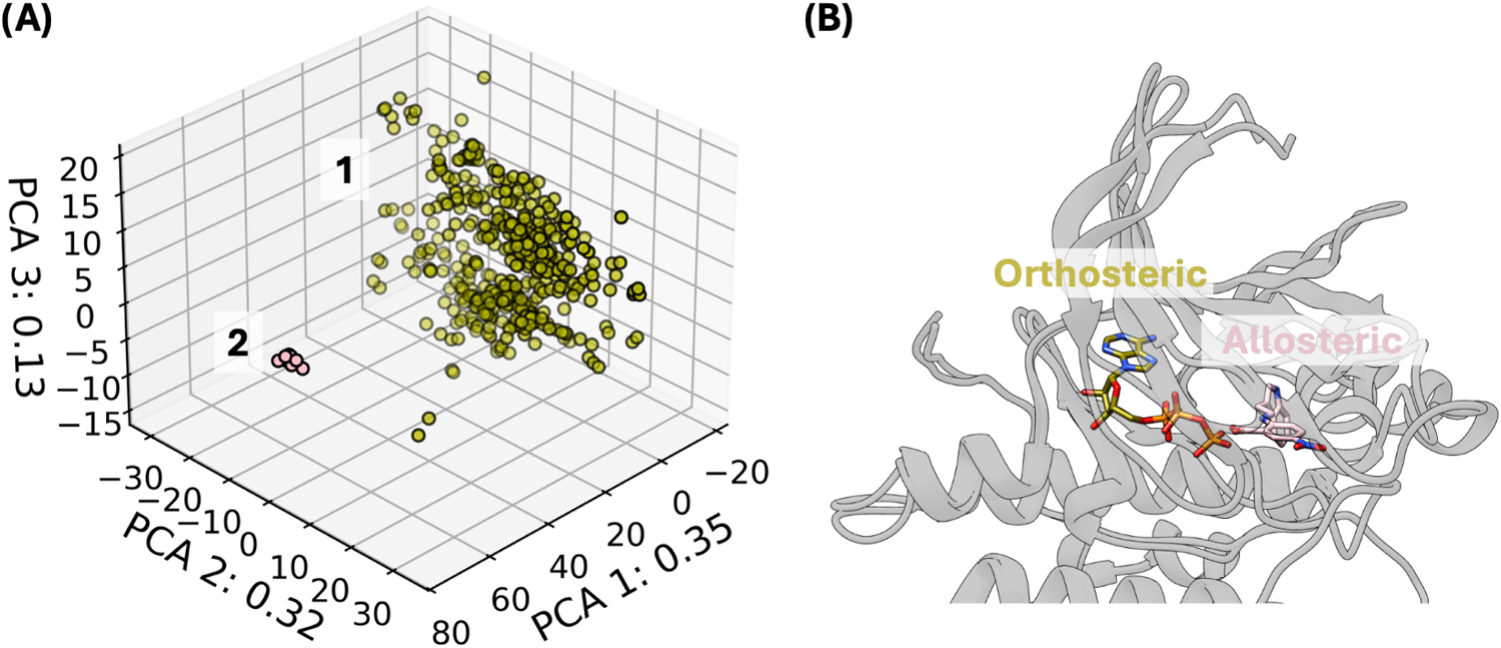
Ligand-only PCA and clustering. (A) The ligand binding location is represented by the residue–ligand minimum distance vector and then input into PCA and HDBSCAN. The data are projected onto the largest PCs that cumulatively contribute to at least 0.75 of the explained variance ratio. This results in two main clusters of orthosteric (c1^L^; yellow) and allosteric ligands (c2^L^; pink). (B) Example structures of orthosteric- and allosteric-bound complexes are colored by ligand-only clustering and represented in sticks.

Another advantage of the minimum distance feature is the straightforward extension to embed the ligand binding location. After removing structures that are *apo* or have multiple of the same ligand bound, we calculate the receptor–ligand minimum distance vector and input this new feature set into PCA and HDBSCAN. After excluding four unclustered ligands as noise, two main clusters emerge: orthosteric binders in the ATP-binding site (c1^L^; yellow) and allosteric binders in the Type III binding site (c2^L^; pink) (Figure 3). Cross-referencing between receptor-only and ligand-only clustering then reveals the variety of binding modes within the data. In principle, similar binding mode analyses can be performed with other methods when extended to include ligand or pocket-based features.^80,81^

The exploration of the receptor-only and ligand-only representation spaces is then leveraged to a couple of docking-related tasks downstream as well. First, we reconstruct missing loop regions using *conformation-based loop modeling*. This method uses nearby conformations in receptor-only space as template structures for remodeling and is detailed in the methods section. Next, we develop target-specific benchmarks that evaluate the ability of blind docking methods to predict ligand binding poses in allosteric sites and discriminate between orthosteric and allosteric ligands.

**Figure 4:**
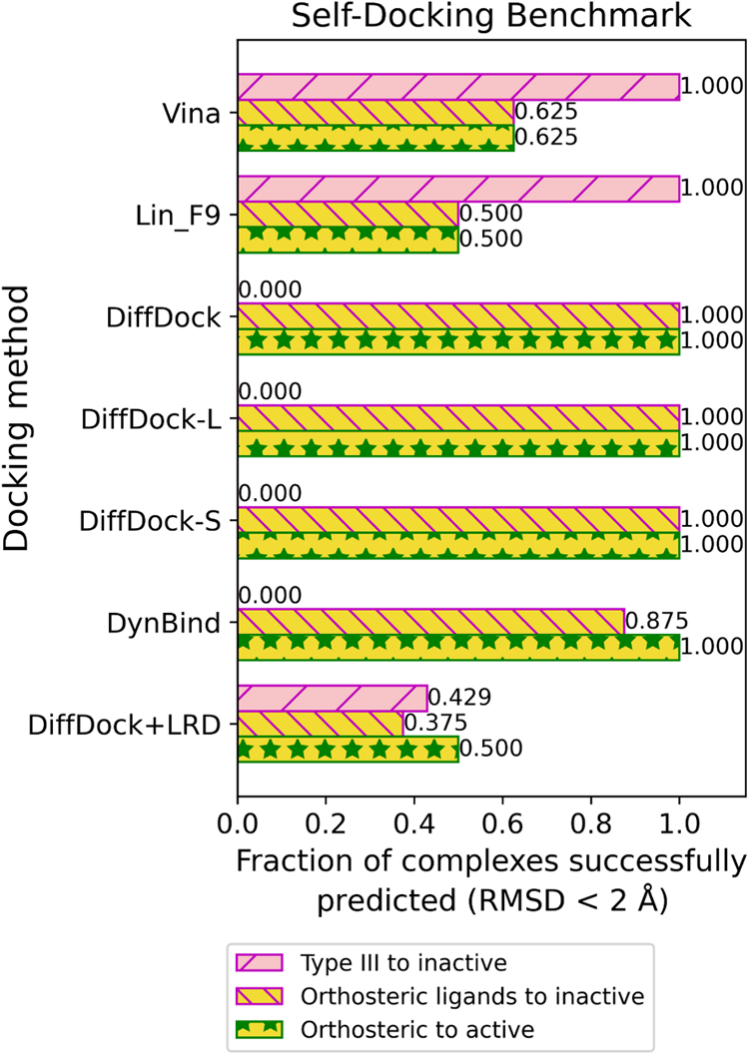
Self-docking benchmark: Fraction of complexes successfully predicted by blind docking models (RMSD < 2 Å). Three binding modes are reported: Type III bound to the inactive state (c2^L^–c1^R^; *n* = 7; pink with purple hashes), orthosteric ligands to the inactive state (c1^L^–c1^R^; *n* = 8; yellow with purple hashes), and orthosteric ligands to the active state (c1^L^–c3^R^; *n* = 8; yellow with green stars). The binding mode is defined by the ligand binding location clustering observed in Figure 3A and receptor conformation clustering in Figure 2A. The identities of the complexes can be found in Table S4. If any pose generated from a specific receptor–ligand pair meets the criteria, then the prediction is marked as successful. LRD: Local Re-Docking.

We create the self-docking benchmark, which assesses the capability of docking methods to accurately predict the pose when given the original receptor conformation and group results depending on the binding mode (Figures 2A and 3A). We select 7 complexes from each binding mode plus the native orthosteric substrate, ATP, and ensure that there is no leakage of the identities of these complexes in the training set (Table S4). We report the fraction of complexes with at least one successfully predicted pose with RMSD < 2 Å to the reference crystal structure (Figure 4). The deep learning blind docking methods are able to predict the orthosteric ligands but are all unable to predict the Type III binding mode.^11−13^ Interestingly, we observe that traditional docking performs best when predicting the Type III binding mode but has more limited success predicting the orthosteric binding mode compared to deep learning docking. Similarly, a combination of deep learning and traditional docking, DiffDock, followed by Lin_F9^8^ Local Re-Docking (DiffDock + LRD), improves the capability of DiffDock to predict the Type III binding pose but loses accuracy with orthosteric binders. These results on Type III binders are consistent with the observations by Yu et al., where improved performance is observed when deep learning docking performs the role of pocket searching and traditional docking as pocket refinement.^16^

We create a cross-docking benchmark to mimic a real-world screening scenario by introducing a time-split, as illustrated in Figure S9. We select receptor structures prior to the determination of the allosteric complexes (8/2/2021) and then perform PCA and HDBSCAN clustering (Figure S5 boxed; Table S5). We once again distinguish 4 clusters and define 3 states: inactive (purple; c1^R-TS^), intermediate (cyan; c2^R-TS^), and active (green and red; c3^R-TS^ and c4^R-TS^). We then take a few receptors from each state and dock the compounds as labeled by their binding mode (Table S4). This benchmark assesses the capability of blind docking methods to prospectively predict and discriminate between the binding modes depending on the given receptor state.

In this benchmark, we do not use the gold standard docking metric, RMSD < 2 Å, because rigid-receptor blind docking methods encounter difficulty with exactly reproducing the ground-truth pose due to clashes in the Type III binding site of the provided time-split receptor. RMSD < 2 Å is more attainable and appropriate for the self-docking benchmark, orthosteric docking, or known-pocket docking when the binding site is often preorganized. Instead, we use a checkpoint to achieve RMSD < 2 Å, the ligand–centroid distance <5 Å metric following all-Cα structural alignment with the crystal structure, and report the fraction of complexes that meet these criteria. This metric quantifies whether the ligand pose is predicted in a site that is spatially similar to the reference crystal structure and indicates the frequency with which these methods sample these sites. Nonetheless, success at this checkpoint is still valuable to SBDD. For example, in our previous work, an accurate blind docking method was needed to suggest targetable residues for mutagenesis and support the identification of the binding site of a hit compound with an uncompetitive mode of inhibition.^79^ To this aim, the ligand–centroid distance metric is appropriate for examining docking performance and guides future directions.

**Figure 5:**
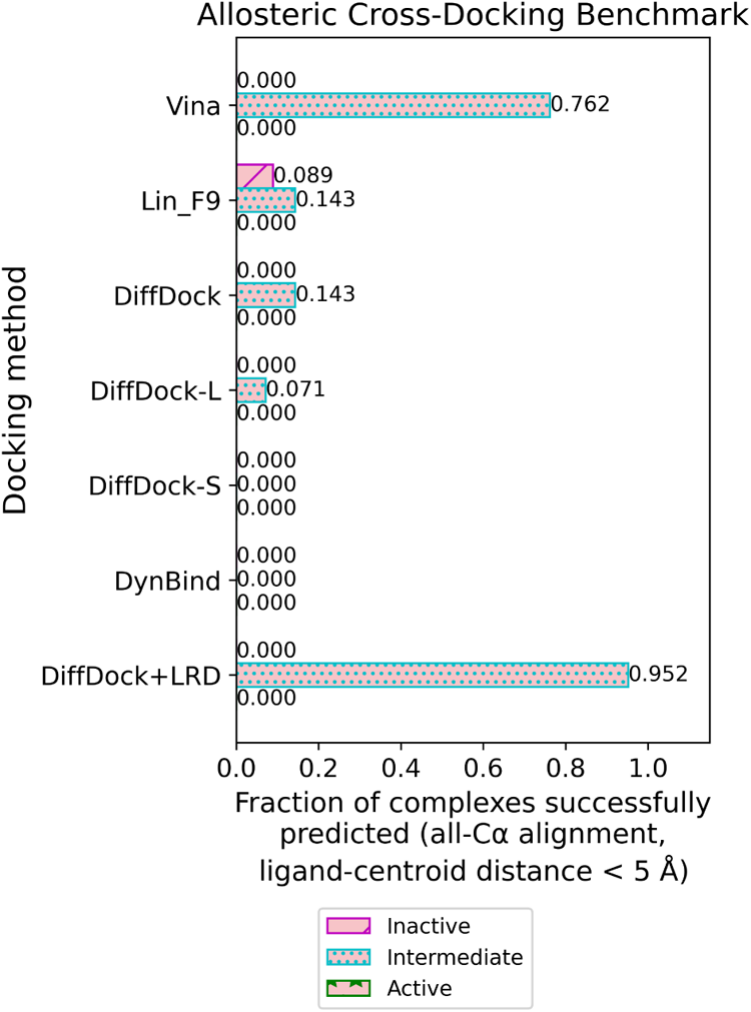
Allosteric cross-docking benchmark: Fraction of complexes successfully predicted by blind docking models (all-Cα alignment, ligand-centroid distance <5 Å). Each Type III ligand (pink) is docked to a set of time-split receptor conformations: inactive (c1^R-TS^; *n* = 8; purple hash), intermediate (c2^R-TS^; *n* = 3; cyan dots), and active (c3^R-TS^; *n* = 8; green stars). The binding mode is defined by the ligand binding location clustering observed in Figure 3A and Table S4, and receptor conformation clustering in Figure S5 boxed and Table S5. If any pose generated from a specific receptor–ligand pair meets the criteria, then the prediction is marked as successful. LRD: Local Re-Docking.

The cross-docking results indicate that we can sample the orthosteric ligand pose regardless of receptor conformation or method and that deep learning docking can do this more reliably than traditional docking (Table S6). In contrast, stricter requirements are needed to sample the Type III ligand pose (Figure 5). Here, the ligands should be docked to the intermediate and ANS–bound receptor conformation (c2^R-TS^), and methods involving traditional docking are more likely to sample this allosteric site compared to purely deep learning methods. A combination of approaches, DiffDock + LRD, samples this mode best when given c2^R-TS^. These results are consistent with the broadly accepted notion that traditional docking performs best when given a structure that has been resolved with a ligand bound in the desired binding site.^82−84^

We perform a pocket-matching protocol to compare the targetable interfaces between example receptors, the description of which can be found in the supporting methods (Figure S10A). We note that these cross-docking results are consistent with the presence of an orthosteric binding site in all conformations but the absence of a highly ligandable Type III binding site in the active and inactive conformations (Figure S10B,C). A flexible pocket docking method, DynamicBind, can be promising in predicting ligand-bound poses using unbound/holo protein structures as a receptor.^13^ This method can improve docking performance on structures where the Type III site is at least partially ligandable, as in the inactive conformation, but we are unable to observe this in our case.

Another important aspect is to assess the ranking performance of the scoring function to distinguish the correct poses from false positives. When we only consider the top-1/-5 ranked orthosteric poses to the active and inactive states, traditional docking exhibits slightly worse performance than deep learning docking across both self- and cross-docking benchmarks (Tables S7 and S8). We also observe a decrease in performance when docking orthosteric ligands to the intermediate conformation and, in particular, when selecting the top-1 pose for DiffDock + LRD in cross-docking (Table S8). When docking Type III inhibitors across both benchmarks, DiffDock + LRD exhibits a greater reduction in ranking performance compared to traditional docking (Tables S7 and S9). The drop-off in cross-docking performance can be displayed with a plot of the difference in cluster size (ΔPop) versus the difference in rank (ΔRank) between the correctly predicted pose and the highest rank of the remaining poses.^85−87^ (Figure S11) Data points in the upper left quadrant indicate that the method frequently samples the correct pose and then correctly ranks the poses. Data points in the right half indicate cases where false positives are ranked better than the correct poses and are consistent with the decrease in performance we observe when selecting the top-1/-5 poses. Overall, these results highlight the robustness of the scoring functions to distinguish the orthosteric pose but suggest that an improved scoring function may be needed when a more diverse set of docked poses is generated.

## Conclusions

An exploration of the protein conformational space and the known ligand binding modes is an essential first step in any drug discovery campaign. This case study with CDK2 highlights the utility of MDMR in providing context and visualization for the structural determinants of the conformational ensemble.^88−90^ This data-driven platform surveys ligand binding modes and protein conformations and identifies a unique intermediate receptor conformation in CDK2. The survey guides benchmarking blind docking methods contingent on the ligand binding mode and protein conformation through self- and cross-docking. In the self-docking benchmark, we observe that traditional docking excels at predicting the allosteric binding mode; deep learning docking excels at the orthosteric binding mode; and the combination of deep learning and traditional docking, DiffDock + LRD, improves at the allosteric binding mode at the cost of the orthosteric binding mode. In the prospective-like scenario from the cross-docking benchmark, there are challenges reproducing the allosteric binding pose with active and inactive receptor structures, regardless of the docking method. We do observe that the best allosteric site sampling occurs when using DiffDock + LRD on the intermediate conformation, but those deep learning methods have difficulty sampling the allosteric site. As the field develops and the number of structures grows for this therapeutic mode of inhibition, this platform can be used to test and compare further targets and methods.

Our case study demonstrates the complications of using deep learning blind docking methods for SBDD of allosteric compounds. First, the allosteric pose prediction is observed only when given a receptor conformation that is ligand bound at the allosteric site. In contrast, orthosteric compounds can be docked accurately regardless of conformation or method. This observation highlights the need to identify or generate diverse receptor conformations for docking, where the allosteric binding site is preorganized.^91,92^ Benchmarking and the development of methods that can produce diverse conformations would greatly aid the discovery of allosteric compounds. On the one hand, there exist strategies that do not rely on deep learning like homology modeling^53,93^ and enhanced sampling simulations.^94^ On the other hand, there exists deep learning sampling strategies such as AlphaFold2-based methods that subsample the multiple sequence alignment^95−98^ and those that produce distributions of conformations^99,100^ or reveal cryptic pockets.^101^ Second, deep learning docking methods do not sample allosteric sites well. We note that there are fewer allosterically bound kinase structures and binding data points compared with their orthosteric counterparts (Figure S12). Thus, diffusion-based generative models trained on this data would be more likely to sample the high-density regions and dock ligands to the orthosteric site. This imbalanced data regime begets the need to adapt these generative blind docking models to sample high-fidelity poses from the low-density regions.^102,103^

It is important to acknowledge that this retrospective docking study is biased by past results and does not preclude that these methods or the selection of other conformations could be successful in future campaigns for allosteric binders.^104,105^ These allosteric binding poses are influenced by the receptor structure, and the receptor conformation is biased by the ligands and methods with which it was determined. A prospective study, where a multitude of ligands with diverse chemical properties are screened and are resolved in a multitude of receptor conformations, can assess blind docking methods and reveal the utility of selecting the correct conformation in an unbiased manner.^105^ Furthermore, increasing the number of sampled poses or conformations can also improve the results.

Future work aims to explore other use cases for this representation and platform. For example, this representation can be used as training data for a pose or conformation classifier. Instead of highlighting the distinguishing distances, we can determine structurally conserved residues by selecting low-variance cliques (Figure S13). This method can be extended to study families of related proteins with an appropriate selection of residues through multiple sequence alignments or structural alignment. Other work aims to systematically expand this framework beyond a target-specific task and show whether trends observed in this work are consistent among other allosteric binders to other kinases or proteins.^106^ Improvement of the generalization capabilities of deep learning methods to sample therapeutically relevant protein conformations and predict allosteric binding poses is essential to the SBDD of allosteric compounds. A diverse database of allosteric and peptide binders to the kinome is required to quantitatively assess the preference of allosteric binding modes for certain receptor conformation and establish pocket-specific fine-tuning strategies for generative blind docking models.^107,108^ Lastly, it will also be necessary to assess heterogeneous complex prediction methods that combine receptor fold generation and ligand pose prediction, such as NeuralPlexer,^109^ RoseTTAFold-AllAtom,^110^ or AlphaFold3.^111^ These methods are poised to circumvent the need to select the correct receptor conformation to dock to by generating the complex in full.^112^

## Data Availability

The receptor and ligand data sets, code, and analysis underlying this study are available at https://github.com/echen1214/dist_analy. The docking poses, input topologies, coordinates, and simulation control files are provided at 10.5281/zenodo.13964938.

## Supporting Information Available

Computational methods of the file processing, MD simulations, lDDT pocket matching, and low-variance cliques; tables and figures including the receptor cluster comparisons, lists of structures and ligands used for self- and cross-docking; and results and schematics (PDF)
