# Supplementary material for: Can Deep Learning Blind Docking Methods be Used to Predict Allosteric Compounds?
Source: J Chem Inf Model. 2025 Apr 1;65(7):3737–48. doi: 10.1021/acs.jcim.5c00331 (PMC12004537; doi:10.1021/acs.jcim.5c00331)
Supplement: Supplementary file 1 — ci5c00331_si_001.pdf [file ci5c00331_si_001.pdf]

Supporting Information: Can Deep Learning Blind Docking Methods be used to Predict Allosteric Compounds?

Eric A. Chen[†] and Yingkai Zhang[*,†,‡,¶]

†*Department of Chemistry, New York University, New York, New York 10003, United States*

‡*Simons Center for Computational Physical Chemistry at New York University, New York, New York 10003, United States*

¶*NYU-ECNU Center for Computational Chemistry, Shanghai, Shanghai 200062, China*

E-mail: yingkai.zhang@nyu.edu

Supporting Methods

PDB file processing

We first obtain 453 Protein Data Bank (PDB) entries before 04/13/2023 containing at least one CDK2 structure (UniProt ID P24941) from the RCSB PDB database.[1,2] The CDK2 structure is selected from each entry amounting to 542 structures of CDK2.[3] All waters, ligands are removed, and the non-canonical amino acids are retained. The residues are renumbered to UniProt numbering according to the SIFTs database.[4] These processed structures are then used for the following analyses and docking preparations. The ligands are selected by first separating the heteroatoms from the PDB entry and then excluding ions, small molecules, crystallographic artifacts and non-canonical amino acids.[5] These processed

ligand ligands are then used for the following analyses.

Other representations

For the analysis of the CDK2 structural ensemble, we use ProDy to perform Essential Mode Analysis (EMA) using Cα atomic fluctuations following iterative superimposition as the input to Principal Component Analysis (PCA).[6–8] In this case, we include only residues 1–286 and exclude 14 tailing residues from the highly disordered N-terminal tail segment. We cluster the EMA-projected data points with Hierarchical Density-Based Spatial Clustering of Applications with Noise (HDBSCAN) using $\hat{\epsilon} =$`cluster_selection_epsilon=0.2`.

Cα–Cα distance matrices or contact maps can also be valid or be more informative dependent on your use case. Cα–Cα distance matrices measure the distance between the residue pairs of Cα atoms. Contact maps are binary representations where

$$\text{map} = \begin{cases} 1, & d_{ij}^{\text{shortest}} < 5 \\ 0, & d_{ij}^{\text{shortest}} > 5 \end{cases} \tag{1}$$

Comparison of protein structures with different sequences relies on the observation that evolutionary sequence variation directs the folding constraints of protein structure.[9–12] Selecting residues on their based on their evolutionary information can be useful for comparing structures across different protein families. In this instance, a multiple sequence alignment (MSA) of the protein subfamily is generated using Clustal Omega.[13] The MSA provides the basis for comparing structures by identifying the residues that play a role in the protein structural fold and aligning corresponding residues to one another across different sequences. The identically-, conservatively- and semi-conserved residues of the alignment across CDK1–20 amounts to 93 residues. Other structural alignments, such as the alignment outlined by the Kinase–Ligand Interaction Fingerprints and Structures (KLIFS) database can also be useful.[14,15] This alignment is a manual structural and sequence alignment of 85 residues in the kinase catalytic site. This may allow for more informative comparisons of the kinase catalytic

site across the kinome but at the expensive of excluding other regions. Post-processing methods such as those using persistent spectral graph theory could reduce the need for careful selection of residues or handling of missing residues.[16]

Molecular dynamics simulations

Structures of the CDK2 complexes are taken from the RCSB PDB database (intermediate: 3PXF_A, inactive: 4FKU_A, active: 4FX3_A). Missing residues are modelled with Modeller in Chimera.[17,18] Force field parameters for 60K and 2AN are generated using GAFF2 and the charge parameters are determined using the AM1-BCC charge model.[19]

All simulations are prepared and performed using GPU-enabled AMBER22.03 and the Amber14sb force field.[20,21] The protein is solvated in a cubic box so that the minimum distance between any protein atom and the edge of the box was 1.5 nm. The water molecules are modelled with the TIP3P model.[22] Na+ or Cl- ions are added to neutralize the charge. Hydrogen atoms are constrained using the SHAKE algorithm.[23] Direct nonbonding interactions are cut off at 12, and long-range electrostatic interactions are modeled using the particle-mesh Ewald treatment.[24] Before each simulation, the solvent is first minimized with 15,000 steps of steepest descent and 20,000 steps of conjugate gradient. Then, the whole system is minimized with 15,000 steepest descent and 20,000 steps of conjugate gradient. At this stage three replicas are created with random initial velocities. For each of the replicas the system is heated with weak protein restraints and constant volume from 10K to 298.15K for 1 ps, and then finally equilibrated without protein restraints for 0.1 ps. Each replica is then run under the NPT ensemble for 500 ns with the Monte Carlo barostat set to 1 atm, Langevin thermostat set to 300 K and integration time step of 2 fs. 5,000 samples are taken from each simulation for a total of 15,000 samples for each complex. All trajectory processing and acquisition of R1 and R3 distances was performed using CPPTRAJ.[25]

Docking pose clustering and ranking analysis

We follow the cluster analysis as described in the AutoDock User Guide 4.2 for each docking method.[26,27] In brief, the top ranked pose is taken as the defining seed for the first cluster. Then progressively iterate down the ranked list and calculate the heavy-atom Root Mean Squared Deviation (RMSD). If RMSD of the sampled pose to the seed below a threshold (0.5) then the pose is added to the cluster. If not, the pose is assigned as its own cluster. Each cluster is assigned a rank, which corresponds to the best rank of the cluster, and a population.

To analyze ranking results, we plot the difference in cluster size (ΔPop) versus the different in rank (ΔRank) between the correctly predicted pose (ligand–centroid distance <5 Å metric following all-Cα structural alignment with the crystal structure) and the best ranked of the remaining predicted poses.

lDDT pocket matching

AlphaSpace and AlphaSpace 2.0 are geometry-based mappings of protein binding pockets.[28,29] These methods rely on calculating Voronoi diagrams and then placing alpha-atoms at the vertices. These alpha-atoms can be considered a theoretical ligand atom whose properties reflect a docked fragment. AlphaSpace 2.0 clusters the alpha-atoms into a β-cluster, a pseudomolecular representation of the pockets comprised of β-atoms. This representation returns a β-space and β-score which reflects the volume and the maximum theoretical docking score respectively. In this case, the docking score is calculated using the Vina scoring function.

To match pockets between structures and compare binding sites, AlphaSpace and AlphaSpace 2.0 rely on a structural superimposition first.[28,29]We expand the distance matrix representation and detach from requiring superimposition or identical structures by implementing a pocket matching protocol inspired by the local Distance Difference Test (lDDT).[30] This aims to match pockets depending on how similar the local environment is to the a ref-

erence structure.

Given a reference and a sample structure, we use AlphaSpace 2.0 to generate a pocket mapping of each protein surface and then calculate a residue–pocket shortest distance vector for each pocket i, L_i. This vector is a representation of the local environment of the pocket. Next, we calculate a pairwise lDDT-based matching score between the reference and sample residue–pocket vectors. Using a max distance of 15 Å, we calculate the fraction of distances differences below a threshold. The lDDT match score is the average of four fractions computed using the thresholds 0.5 Å, 1 Å, 2 Å and 4 Å. The sample pockets are then matched to the reference pockets depending on the maximum lDDT match score. (**Figure S10A**)

Low variance cliques

We determine low variance cliques to describe a set of structurally conserved residues over our dataset. The NetworkX package is used to determine the maximally sized residue cliques (complete subgraph) whose pairwise inter-residue variances are below a range of thresholds (**Figure S13A**).[31] The combined plot of the maximal clique size and number of max cliques as the threshold increases is used to select the threshold cutoff and define the clique (**Figure S13B**). In practice, we select the threshold cutoff where the number of max cliques are low. The resulting clique residues form a fully-connected graph whereby the shortest distances between them are minimally variant. These cliques can be a valuable data-driven approach for an unbiased selection of residues to perform superimposition.

Supporting Tables

Table S1: Cluster comparison of **Figure 2** with the Möbitz kinase classification. [a, 32]

DFG-motif	Cluster	$c1^R$	$c2^R$	$c3^R$	$c4^R$
DFGin	Active	0	0	102	2
	DFG-active αC-out	0	0	0	0
	FG-down	0	0	1	0
	FG-down αC-out	178	0	0	0
	G-down	0	0	0	0
	G-down αC-out	53	5	0	0
DFGout	DFG-flipped	0	0	0	0
	DFG-out type 2	0	0	0	0
	A-under-P BRAF	0	0	0	0
	A-under-P FMS	0	0	0	0
	A-under-P IGF1R	0	0	0	0
	A-under-P MET	0	0	0	0
	Other	3	0	0	0
	Disordered	8	0	0	0

[a] Möbitz uses pseudo-torsion (4 consecutive Cα atoms) angles involving the DFG motif and distances to the αC-helix Glu.

Table S2: Cluster comparison of **Figure 2** with the Ung *et al.* kinase classification.[a, 33]

Spatial Group[b]	$c1^R$	$c2^R$	$c3^R$	$c4^R$
CIDI	0	0	35	0
CIDO	0	0	0	0
CODI	161	5	0	0
CODO	0	0	0	0
ωCD	0	0	0	0

[a] Ung *et al.* developed a random forest classifier on features such as the DFG-Asp and DFG-Phe Cα–Cβ vectors, and structural descriptors of the αC-helix (vectors, distances and angles involving the αC-helix Glu).

[b] CICI: αC-helix in–DFG-in; CIDO: αC-helix in–DFG-out; CODI: αC-helix out–DFG-in; CODO: αC-helix out–DFG-out ; ωCD: DFG-intermediate

Table S3: Cluster comparison of **Figure 2** with the Modi and Dunbrack kinase classification. [a, 34,35]

Spatial Group	Cluster	$c1^R$	$c2^R$	$c3^R$	$c4^R$
DFGin	BLAminus	9	0	127	4
	BLAplus	0	0	0	0
	ABAminus	0	0	1	1
	BLBminus	79	6	0	0
	BLBplus	19	0	0	0
	BLBtrans	123	0	0	0
	None	14	0	1	1
DFGout	BBAminus	0	0	0	0
	None	0	0	1	0
DFGinter	BABtrans	0	0	0	0
	None	0	0	0	0

[a] Modi and Dunbrack use conserved N-terminal domain distances between the β3 sheet and αC-helix to the DFG-Phe-Cζ, and backbone dihedral angles of the XDF residues and the DFG-Phe first side chain torsion

Table S4: Assign a binding mode to ligands bound to a PDB structure.[a]

Binding Mode	PDB_chain	Ligand_resnum
Type III bound to inactive state ($c2^{L}$–$c1^{R}$; n=7)	7RWF_A, 8FOW_A, 8FP0_A	7TW_302, 7TW_304, 7TW_304
	7RXO_A	80E_302
	7S4T_A	88O_302
	7S7A_A	8FI_302
	7S84_A	8IL_301
	7S85_A	8IQ_303
	7S9X_A	8KF_301
Orthosteric ligands to inactive state ($c1^{L}$–$c1^{R}$; n=8)	1B38_A	ATP_381
	1GIH_A	1PU_501
	1GIJ_A	2PU_501
	2BHE_A	BRY_1299
	2R3R_A	6SC_501
	2VTR_A	LZB_1299
	4FKG_A	4CK_300
	4FKI_A	09K_301
Orthosteric ligands to active state ($c1^{L}$–$c3^{R}$; n=8)	1JST_A	ATP_300
	2C5V_C	CK4_1297
	3BHT_A	MFR_299
	3BHU_A	MHR_299
	4BCM_C	T7Z_1295
	4CFW_C	SQ9_1297
	7ACK_C	R7B_301
	7KJS_A	WG1_301

[a] Receptor and ligand cluster definitions are defined by **Figure 1A** and **Figure 2A**, respectively.

Table S5: Receptor structures from the time split (R-TS) used in the cross-docking benchmark.[a]

Receptor cluster	PDB_chain
Inactive ($c1^{R\text{-}TS}$; purple; n=8)	1B38_A, 1GIH_A, 1GIJ_A, 2BHE_A, 2R3R_A, 2VTR_A, 4FKG_A, 4FKI_A
Intermediate ($c2^{R\text{-}TS}$; cyan; n=3)	3PXF_A, 3PXQ_A, 4EZ7_A
Active ($c3^{R\text{-}TS}$; green, n=8)	1JST_A, 2C5V_C, 3BHT_A, 3BHU_A, 4BCM_C, 4CFW_C, 7ACK_C, 7KJS_A

[a] Receptor cluster definitions are defined by **Figure S5** boxed.

Table S6: Orthosteric cross-docking benchmark: Fraction of complexes that are successfully predicted by blind docking models (all-Cα alignment, ligand-centroid distance <5 Å)[a,b]

Binding Mode	Orthosteric ligands to inactive state ($c1^L$–$c1^R$; n=8)			Orthosteric ligands to active state ($c1^L$–$c3^R$; n=8)		
Receptor conformation	I	Mid	A	I	Mid	A
Vina	0.929	0.667	0.984	0.873	0.708	1.000
Lin_F9	0.911	0.958	1.000	0.952	0.792	1.000
DiffDock	1.000	0.958	1.000	1.000	1.000	1.000
DiffDock-L	1.000	1.000	1.000	1.000	1.000	1.000
DiffDock-S	1.000	1.000	1.000	1.000	1.000	1.000
DynamicBind	1.000	1.000	1.000	1.000	1.000	1.000
DiffDock+LRD[c]	1.000	0.958	1.000	0.984	0.958	1.000

[a] Each orthosteric ligand is docked to a time-split set of receptor conformations where I=inactive (purple; $c1^{R\text{-}TS}$; n=8), Mid=intermediate (cyan; $c2^{R\text{-}TS}$; n=3), active (green; $c3^{R\text{-}TS}$; n=8). The ligand binding mode is defined by the clustering observed in **Figure 3A**, **Table S4** and receptor conformation is defined by the clustering in **Figure S5** boxed, **Table S5**.

[b] If any pose generated from a specific receptor–ligand pair meets the criteria, then the prediction is marked as successful.

[c] LRD: Local Re-Docking

Table S7: Self-docking benchmark with ranked poses: Fraction of the top n poses per complex that are successfully predicted by blind docking models (RMSD <2 Å).[a]

Binding Mode	Type III bound to inactive state ($c2^L$–$c1^R$; n=7)		Orthosteric ligands to inactive state ($c1^L$–$c1^R$; n=8)		Orthosteric ligands to active state ($c1^L$–$c3^R$; n=8)	
Top n	5	1	5	1	5	1
Vina	1.00	1.00	0.38	0.38	0.50	0.25
Lin_F9	1.00	1.00	0.38	0.00	0.50	0.50
DiffDock	0.00	0.00	1.00	1.00	0.88	0.88
DiffDock-L	0.00	0.00	1.00	1.00	1.00	1.00
DiffDock-S	0.00	0.00	1.00	0.88	1.00	0.88
DynamicBind	0.00	0.00	0.88	0.75	1.00	0.88
DiffDock+LRD[b]	0.43	0.14	0.38	0.12	0.50	0.50

[a] The binding mode is defined by the clustering observed in **Figure 3A** and receptor conformation is defined by the clustering in **Figure 2A**. The identities of complexes can be found in **Table S4**.

[b] LRD: Local Re-Docking

Table S8: Orthosteric cross-docking benchmark with ranked poses: Fraction of the top n poses per complex that are successfully predicted by deep learning models (all-Cα alignment, ligand-centroid distance <5 Å)[a,b]

Binding Mode	Orthosteric ligands to inactive state ($c1^L$–$c1^R$; n=8)						Orthosteric ligands to active state ($c1^L$–$c3^R$; n=8)					
Receptor conformation	I		Mid		A		I		Mid		A	
Top n	5	1	5	1	5	1	5	1	5	1	5	1
Vina	0.91	0.84	0.42	0.33	0.98	0.97	0.87	0.86	0.62	0.50	1.00	0.95
Lin_F9	0.82	0.77	0.88	0.62	0.98	0.95	0.95	0.84	0.71	0.62	1.00	1.00
DiffDock	1.00	1.00	0.96	0.83	1.00	1.00	1.00	1.00	1.00	0.92	1.00	1.00
DiffDock-L	1.00	1.00	1.00	1.00	1.00	1.00	1.00	1.00	1.00	1.00	1.00	1.00
DiffDock-S	1.00	1.00	1.00	1.00	1.00	1.00	1.00	1.00	1.00	1.00	1.00	1.00
DynamicBind	1.00	1.00	1.00	1.00	1.00	1.00	1.00	1.00	1.00	1.00	1.00	1.00
DiffDock+LRD[c]	1.00	0.98	0.88	0.67	1.00	0.95	0.97	0.92	0.96	0.83	1.00	1.00

[a] Each orthosteric ligand is docked to a time-split receptor conformations where I=inactive ($c1^{R\text{-}TS}$; n=8), Mid=intermediate ($c2^{R\text{-}TS}$; n=3), A=active ($c3^{R\text{-}TS}$; n=8). The ligand binding mode is defined by the clustering observed in **Figure 3A**, **Table S4** and receptor conformation is defined by the clustering in **Figure S5** boxed, **Table S5**.

[b] If any top-n pose generated from a specific receptor–ligand pair meets the criteria, then the prediction is marked as successful.

[c] LRD: Local Re-Docking

Table S9: Allosteric cross-docking benchmark with ranked poses: Fraction of the top n poses per complex that are successfully predicted by blind docking models (all-Cα alignment, ligand-centroid distance <5 Å) [a,b]

Binding Mode	Type III bound to inactive state ($c2^L$–$c1^R$; n=7)					
Receptor conformation	I		Mid		A	
Top n	5	1	5	1	5	1
Vina	0.00	0.00	0.67	0.43	0.00	0.00
Lin_F9	0.05	0.02	0.05	0.05	0.00	0.00
DiffDock	0.00	0.00	0.00	0.00	0.00	0.00
DiffDock-L	0.00	0.00	0.00	0.00	0.00	0.00
DiffDock-S	0.00	0.00	0.00	0.00	0.00	0.00
DynamicBind	0.00	0.00	0.00	0.00	0.00	0.00
DiffDock+LRD[c]	0.00	0.00	0.76	0.33	0.00	0.00

[a] Each Type III ligand is docked a set of time-split receptor conformations where I=inactive ($c1^{R\text{-}TS}$; n=8), Mid=intermediate ($c2^{R\text{-}TS}$; n=3), A=active ($c3^{R\text{-}TS}$; n=8). The ligand binding mode is defined by the clustering observed in **Figure 3A**, **Table S4** and receptor conformation is defined by the clustering in **Figure S5** boxed, **Table S5**.

[b] If any top-n pose generated from a specific receptor–ligand pair meets the criteria, then the prediction is marked as successful.

[c] LRD: Local Re-Docking

Supporting Figures

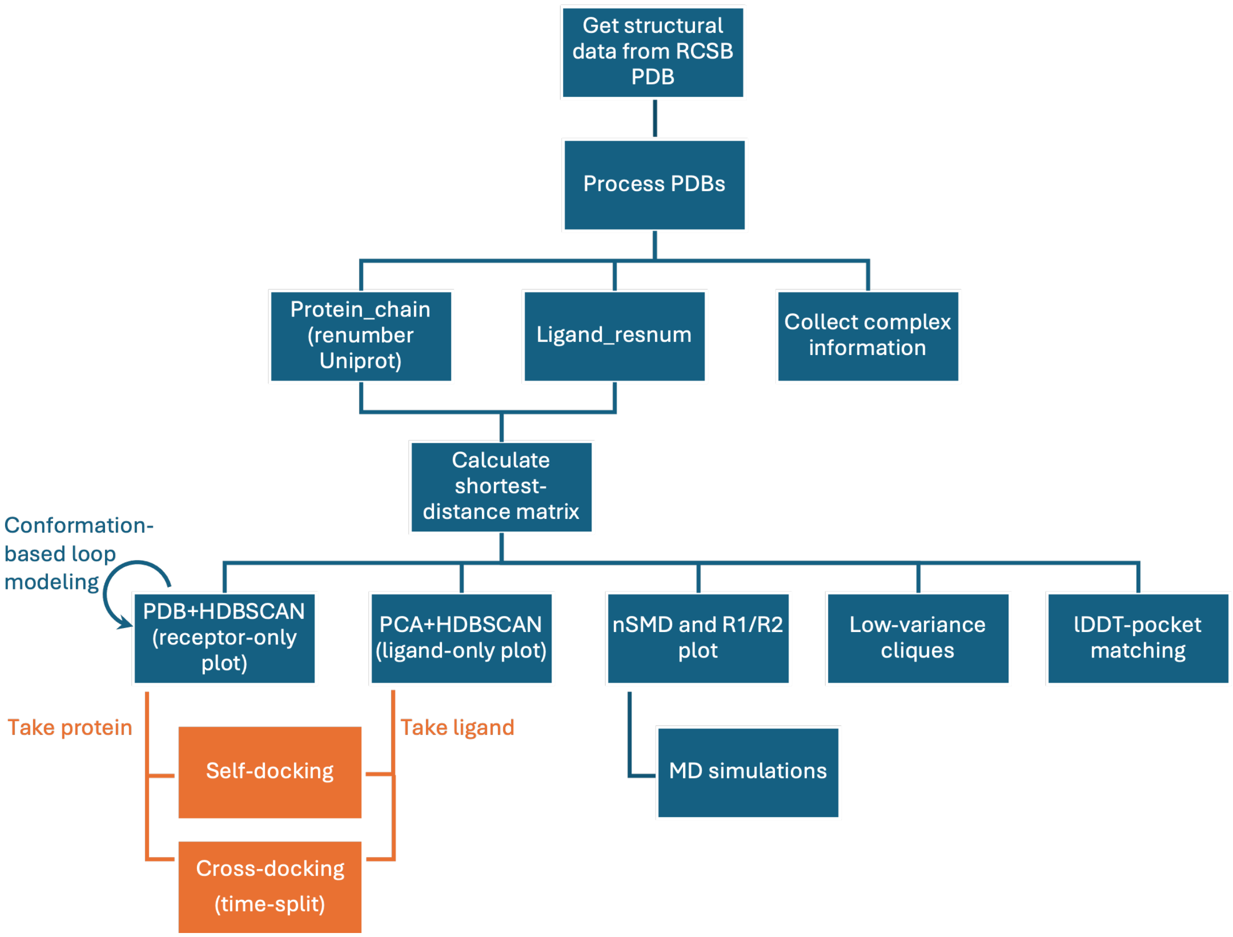

Figure S1: Overall schematic of the workflow and tools offered by this method

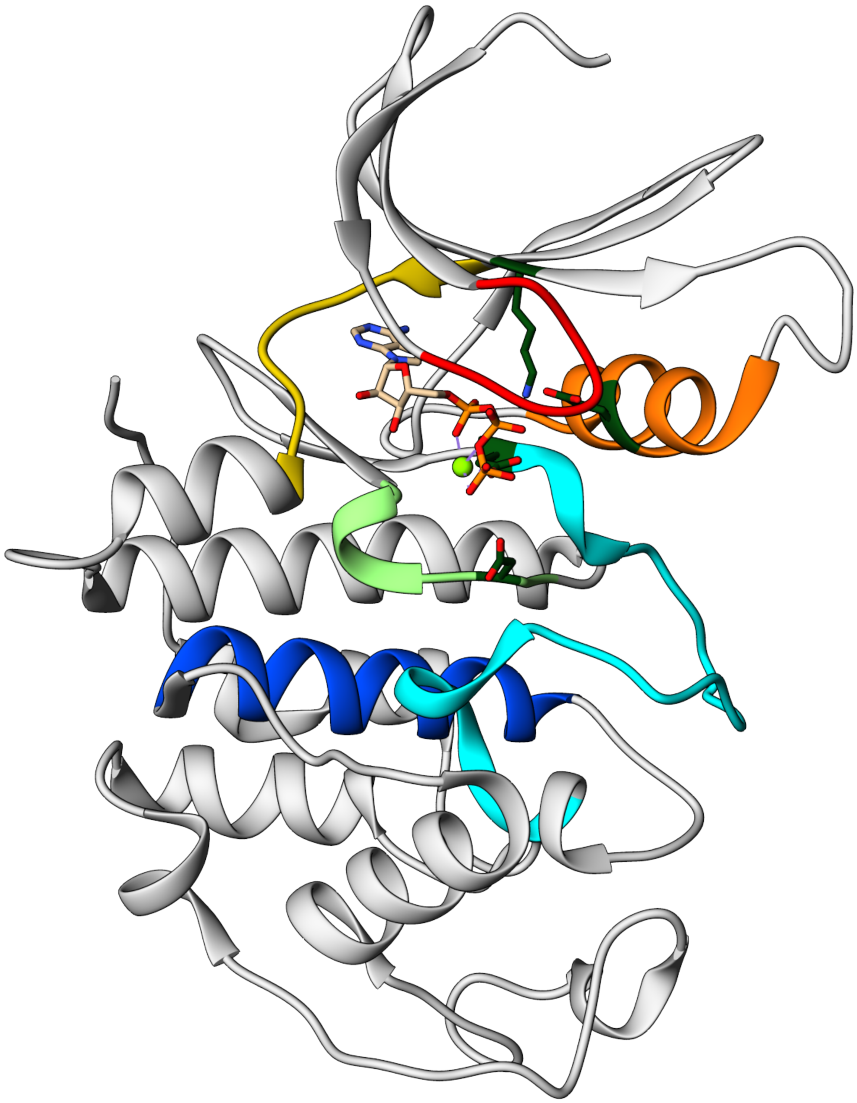

Figure S2: CDK2 Kinase regions using PDBID 1QMZ_A with ATP (*tan* stick) and Mg (*lime* ball). Gly-rich region (12–17) *red*; αC-helix (45–58) *orange*; hinge region (80–87) *yellow*; catalytic loop (125–132) *lime*; KEDD residues (33,51,127,145) *green*; activation segment (146–172) *cyan*; αF-helix (183–197) *blue*

```
All CDK2 [0, 311, 6, 244, 8] 569
├── Cyclin bound [0, 0, 0, 237, 8] 245
│   ├── TPO166 modified [0, 0, 0, 169, 8] 177
│   │   ├── ATP/ADP bound [0, 0, 0, 28, 0] 28
│   │   ├── Other [0, 0, 0, 125, 5] 130
│   │   └── None [0, 0, 0, 16, 3] 19
│   └── None [0, 0, 0, 68, 0] 68
│       ├── ATP/ADP bound [0, 0, 0, 2, 0] 2
│       ├── Other [0, 0, 0, 50, 0] 50
│       └── None [0, 0, 0, 16, 0] 16
├── Other [0, 6, 0, 6, 0] 12
└── None [0, 305, 6, 1, 0] 312
    ├── CYS177 modified [0, 12, 0, 0, 0] 12
    ├── Other [0, 51, 0, 1, 0] 52
    └── None [0, 242, 6, 0, 0] 248
        ├── ATP/ADP bound [0, 2, 0, 0, 0] 2
        ├── Other [0, 236, 6, 0, 0] 242
        └── None [0, 4, 0, 0, 0] 4
```

Figure S3: Binder and mutation cluster makeup of **Figure 2A**.

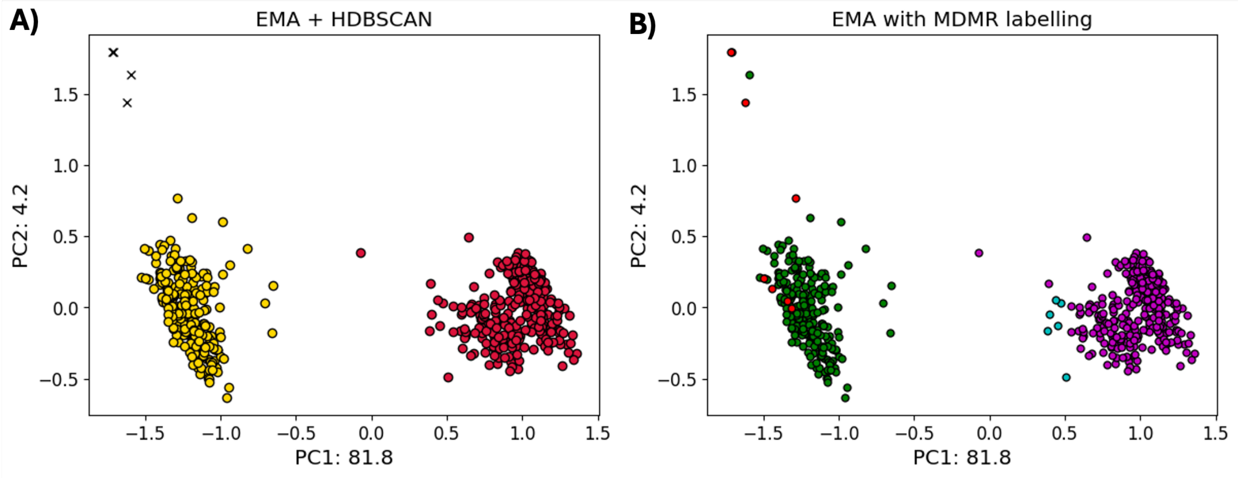

Figure S4: EMA using Cα atomic fluctuations **A)** EMA and HDBSCAN of residues 1–286 for the available CDK2 protein conformations. The yellow clusters represent the active state, while the crimson cluster represents the inactive and intermediate state. Noisy and unclustered data points are represented with an ×. **B)** The cluster labels and coloring from the MDMR approach in **Figure 2A** on the EMA plot. The $c2^R$ intermediate state (cyan) is not distinguished by the EMA plot.

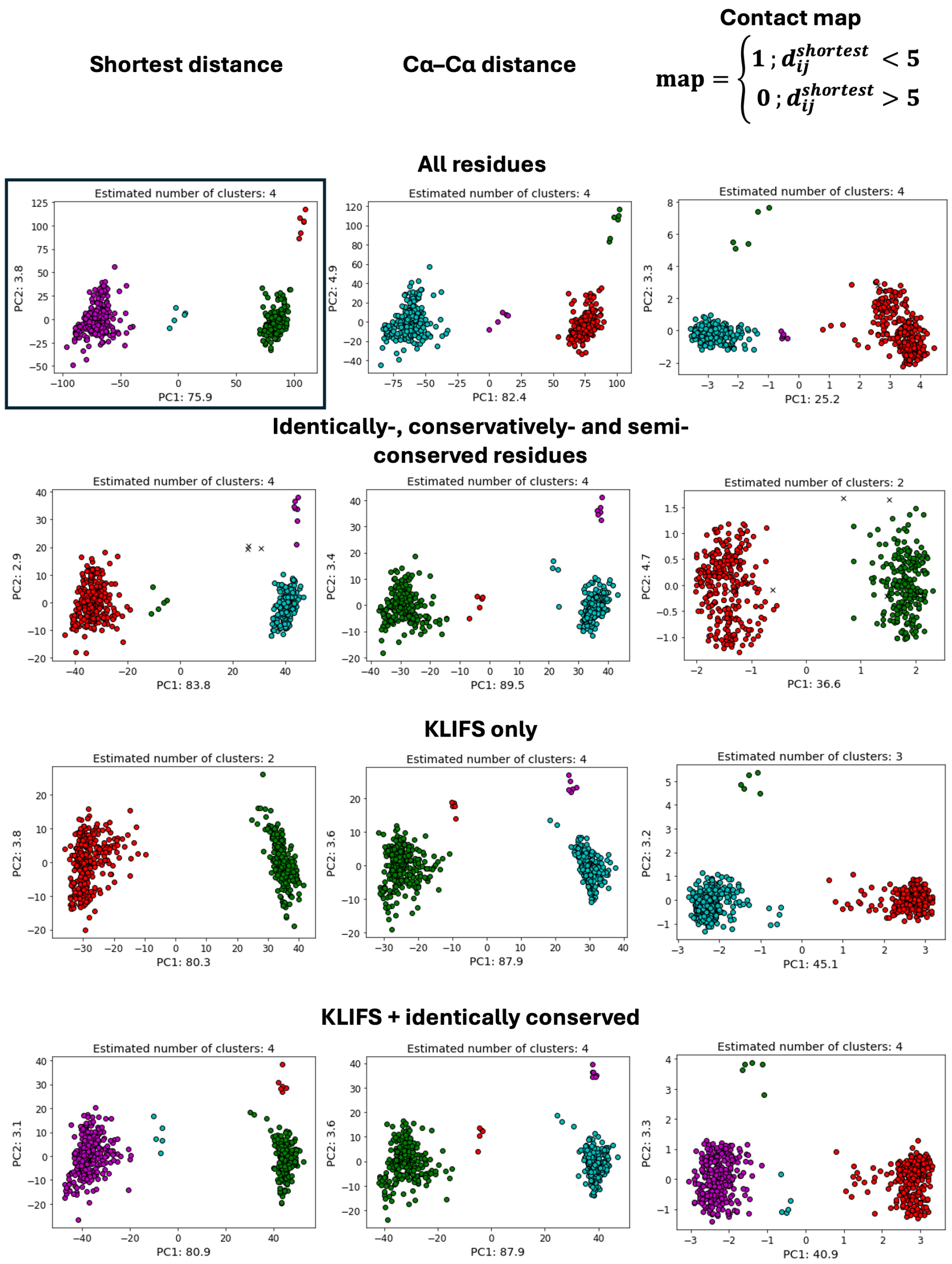

Figure S5: Principal Component Analysis (PCA) and Hierarchical Density-Based Spatial Clustering of Applications with Noise (HDBSCAN) clustering depending on differing residue sets and protein representations. The different residue sets are a) all residues b) identically-, conservatively- and semi-conserved residues following the sequence alignment of CDK1–20 c) KLIFS structural alignment residue set d) union of KLIFS structural alignment set and identically conserved residues (*rows*). For detailed information, see the supporting methods section. The different protein representations are the shortest pairwise distance matrix, Cα-Cα pairwise distance matrix, and contact map (*columns*). The data is projected onto the axes that cumulatively contributed to 0.75 of the explained variance ratio and input into HDBSCAN. We also select PDB entires before 8/2/2021. The box highlights the PCA plot and clustering used to set up the cross-docking experiment

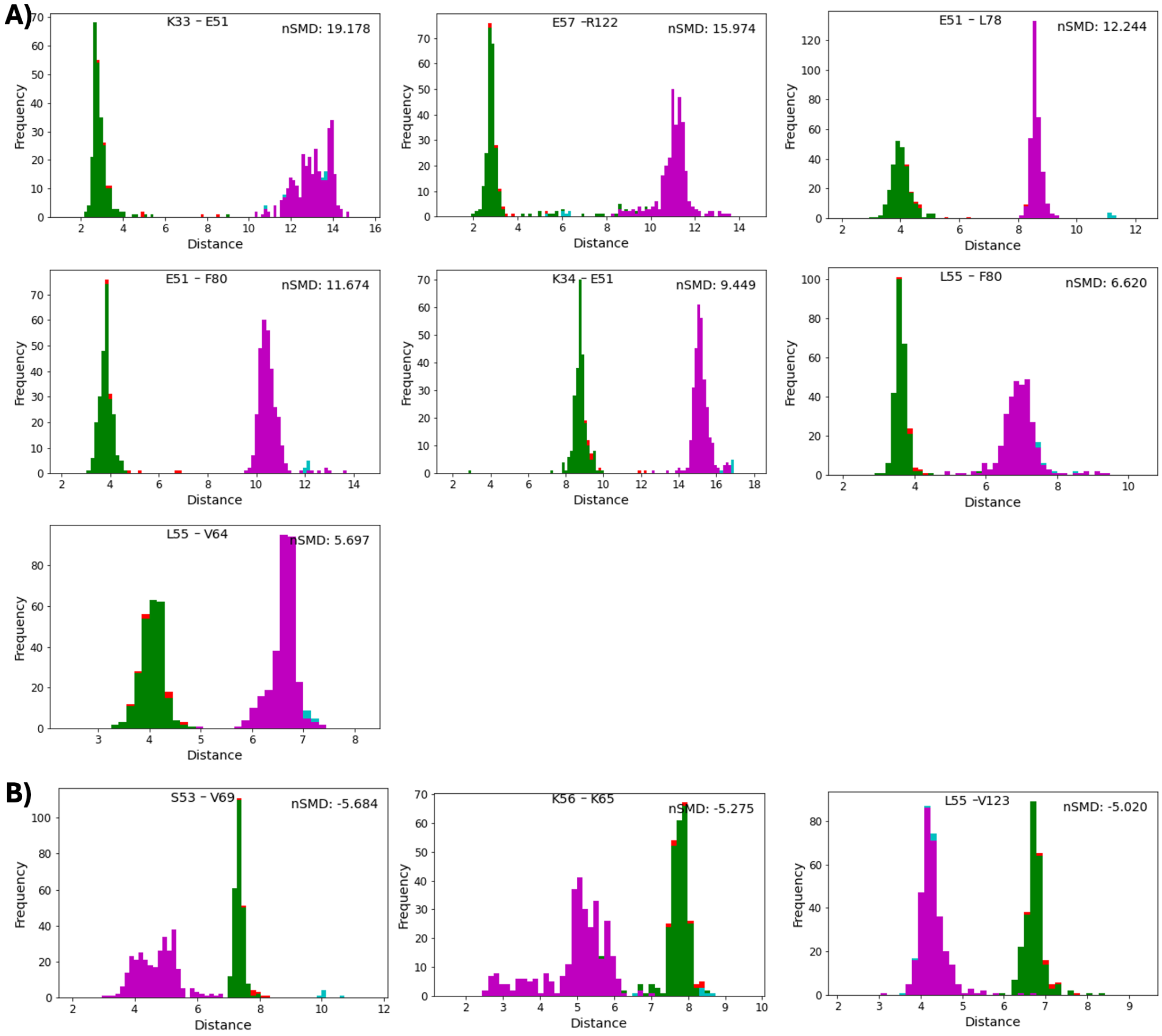

Figure S6: Histograms of the **A)** R3 and **B)** R1 shortest distances and the reported normalized Standardized Mean Difference (nSMD) values. The bar plots are colored by the clustering defined in **Figure 2A**.

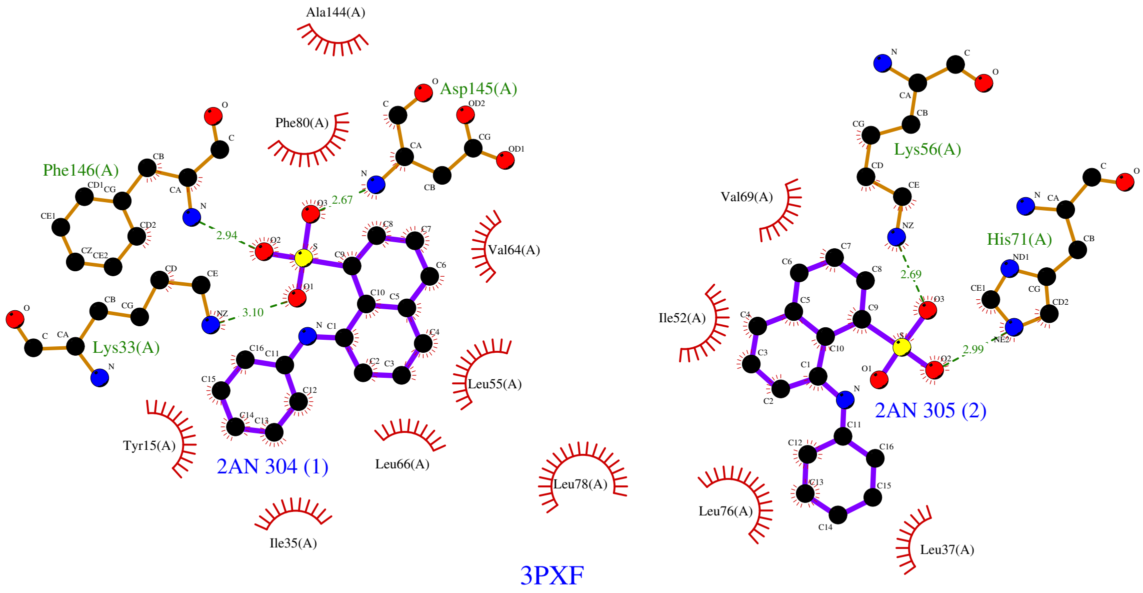

Figure S7: Ligand interaction plot of 3PXF chain A bound by two copies of 8-anilino-1-naphthalene sulfonate (ANS; PDB ligand ID: 2AN).[36,37]

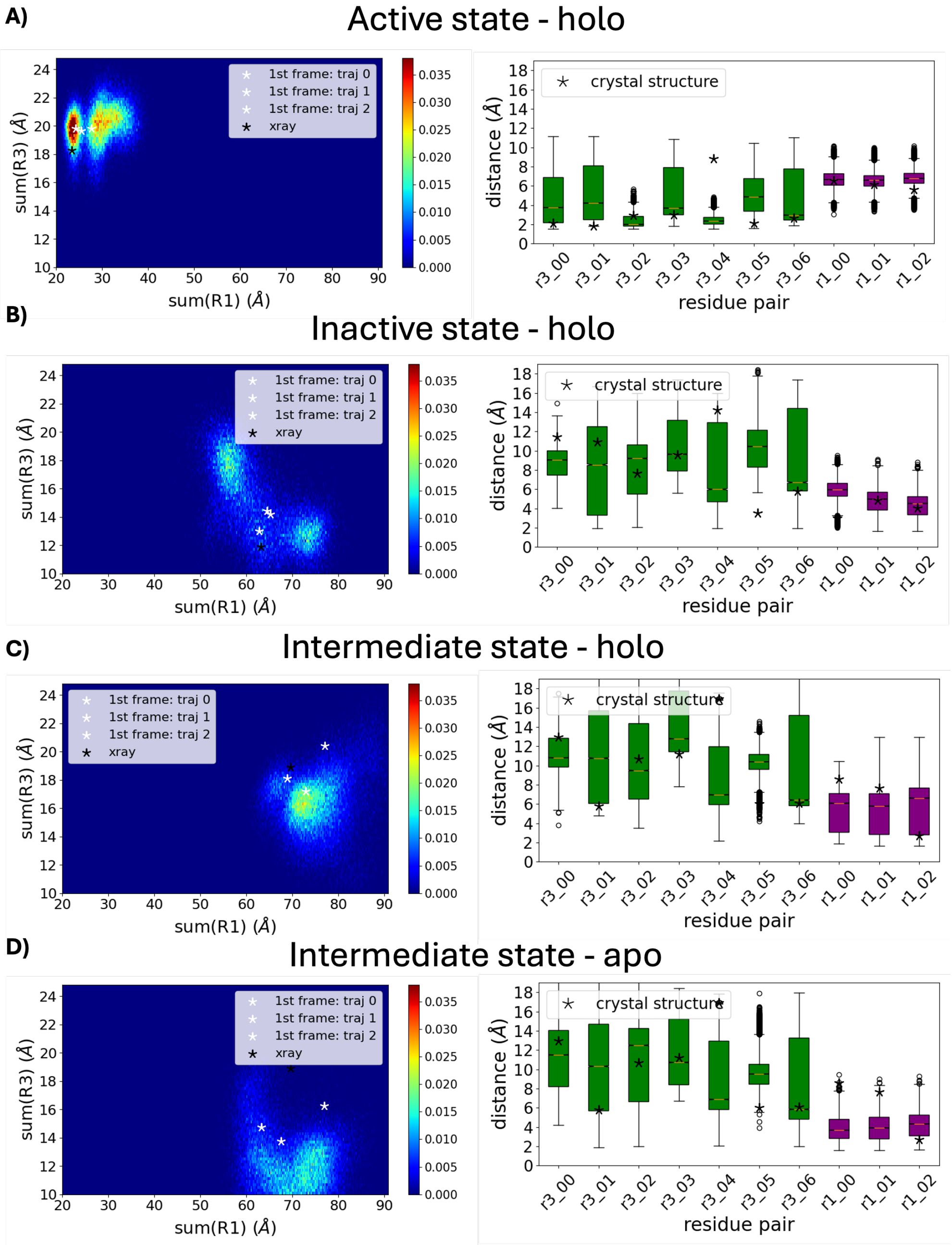

Figure S8: Sum(R2) vs sum(R1) histograms and box-and-whisker plots of MD simulations. A triplicate of 500 ns for each initial condition was run. **A)** Simulation of 4FX3_A bound by 60K **B)** Simulation of 4FKU_A bound by 60K **C)** Simulation of 3PXF_A bound by two copies of 2AN **D)** Simulation of 3PXF_A *apo*. The initial crystal structure position is designated by a black star, and the first frame of each trajectory is reported with a white star. The residue pair label indexing corresponds to **Figure 2B** *below*.

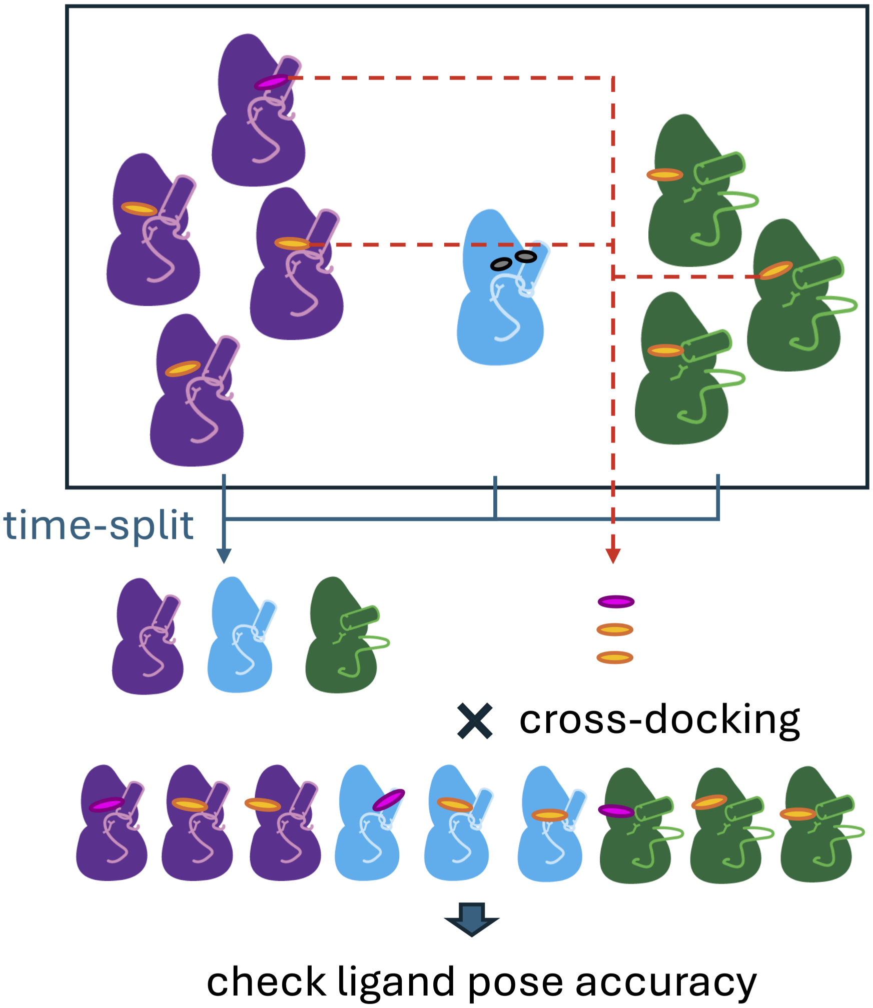

Figure S9: Schematic of the cross-docking benchmark utilized from a time-split. The cross-referencing of the clustering from the ligand-only (binding location) representation space (**Figure 3**) and receptor-only (receptor conformation) (**Figure 2A**) define the binding modes. The binding mode definitions of the ligands are tracked and the ligands docked to the ensemble of selected receptor conformations from the time-split (**Figure S5** boxed). The binding pose is evaluated using ligand–centroid distance from crystal structure following all-Cα structural alignment <5 Å.

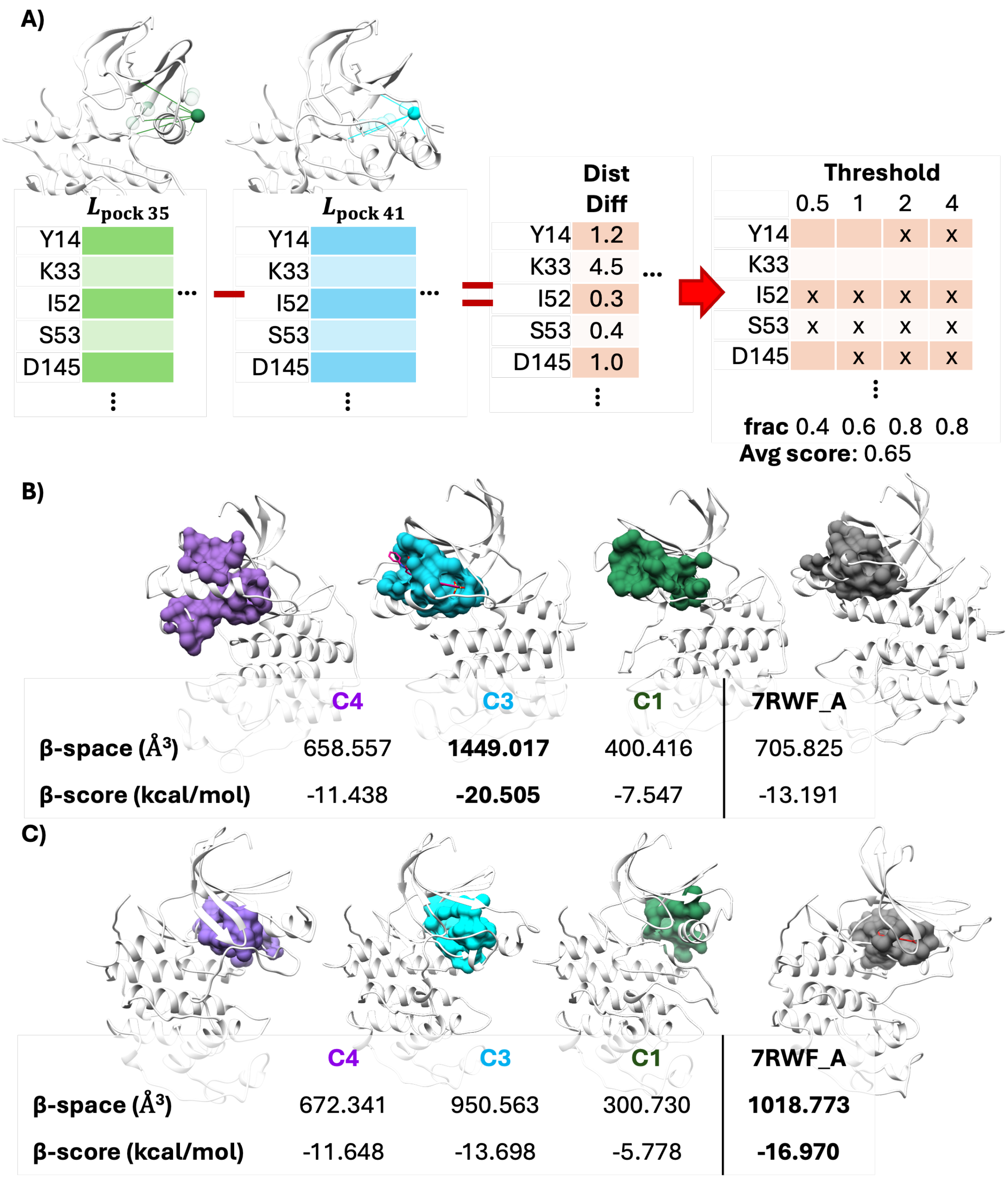

Figure S10: lDDT-based pocket matching. **A)** Schematic of how the lDDT-match score is calculated between pocket 35 of 1QMZ_A and pocket 41 of 3PXF_A. **B)** Match the ANS-occupied pockets of 3PXF_A (c3) to the surface pockets of 4FKU_A (c4), 1QMZ_A (c1) and a example Type III bound structure 7RWF_A. Table of the corresponding β-space and β-scores of the matched pockets. **C)** Match the 7TW-occupied pockets of 7RWF_A to the surface pockets of 4FKU_A (c4), 3PXF_A (c3), and 1QMZ_A (c1). Table of the corresponding β-space and β-scores of the matched pockets. Ligand colored in red. Bolded values indicate the features of the pockets that contact the ligand.

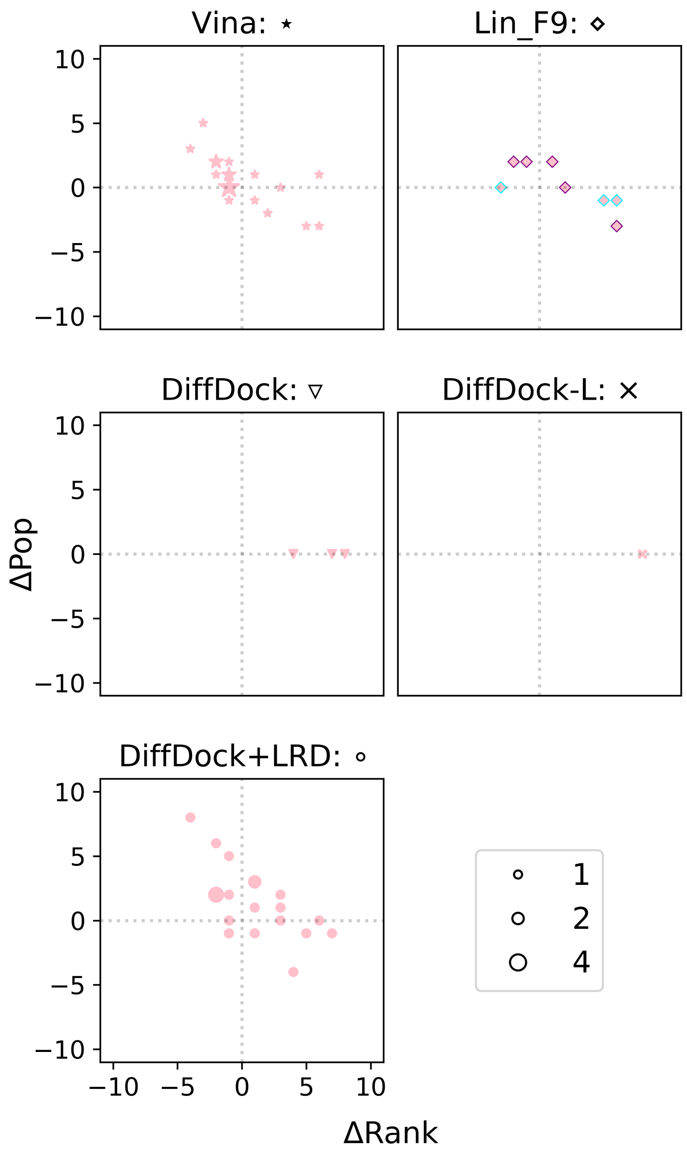

Figure S11: Difference in cluster size (ΔPop) versus difference in rank (ΔRank) between the correctly predicted pose and the best of the remaining predicted poses for the ligands successfully docked (ligand–centroid distance <5 Å metric following all-Cα structural alignment with the crystal structure) from 5 docking methods. The size of each marker represents the number of complexes that fall within each bucket and each method is represented by a different marker. The marker edge colors of the Lin_F9 plot, depict the receptor conformation defined in clustering in **Figure S5** boxed, **Table S5**. All other plots depict the results from the docking only to the intermediate state ($c3^{R\text{-}TS}$)

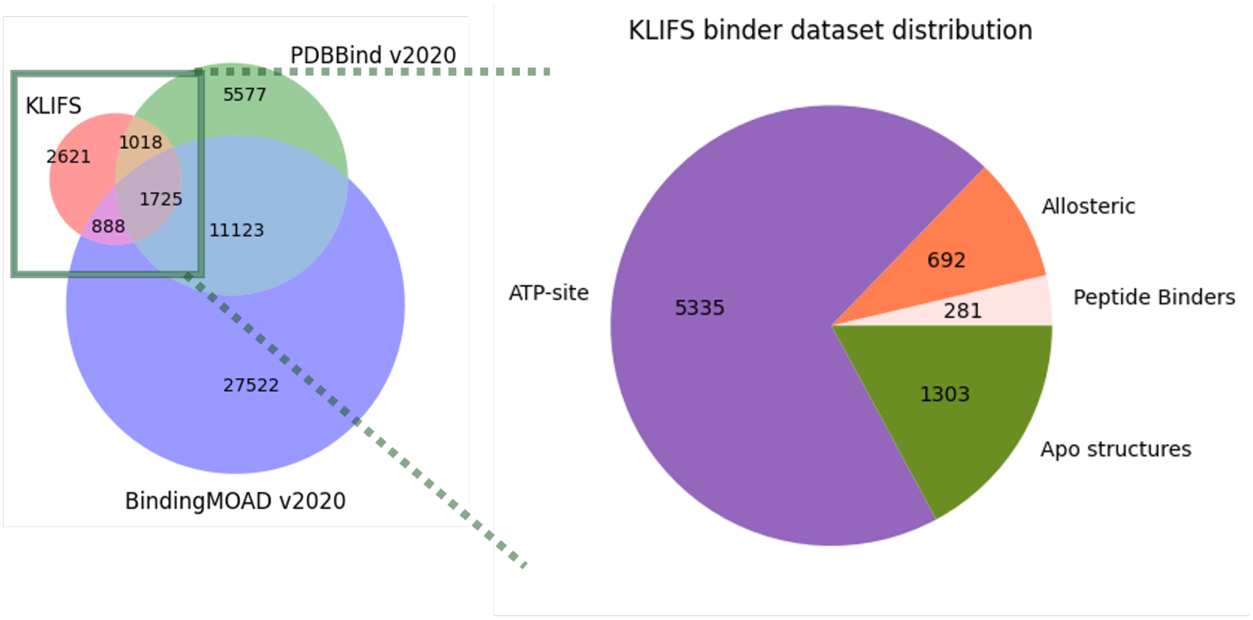

Figure S12: Venn Diagram of complexes represented in the KLIFs database,[14,15] the PDB-Bind v2020,[38] and BindingMOAD.[39,40] Pie chart describing the proportions of competitive vs allosteric vs peptide binders in the KLIFs database.

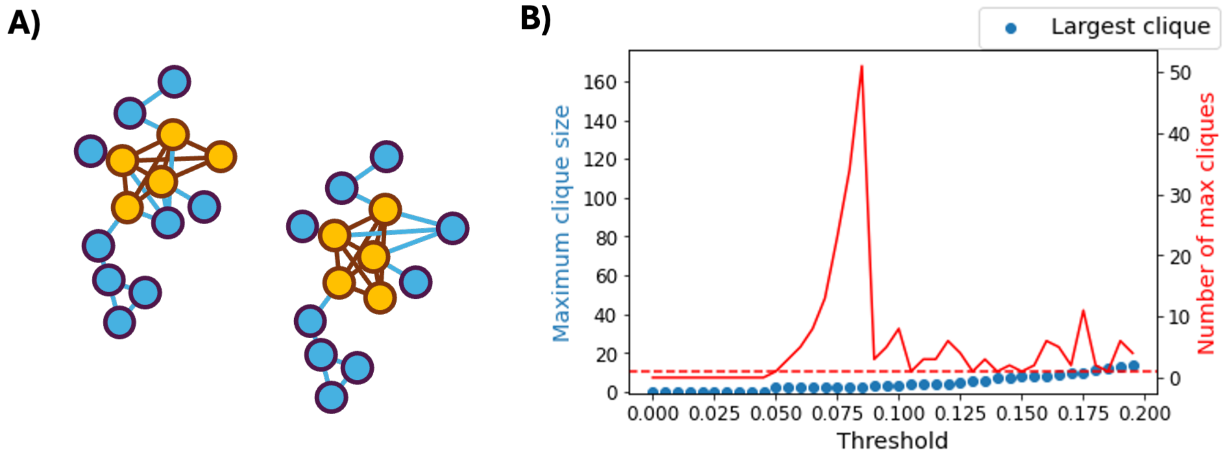

Figure S13: Low-variance clique **A)** Schematic of the definition of a low-variance clique. The nodes depict the residues with edges below a certain inter-residue shorted-distance variance threshold. Highlighted in yellow are a selected clique (complete subgraph). Multiple cliques can be observed for one threshold. **B)** Combined plot of the maximal clique size and number of max cliques as the threshold increases.

References

(1) Burley, S. K. et al. RCSB Protein Data Bank: powerful new tools for exploring 3D structures of biological macromolecules for basic and applied research and education in fundamental biology, biomedicine, biotechnology, bioengineering and energy sciences. *Nucleic Acids Res.* **2021**, *49*, D437–D451, DOI: 10.1093/nar/gkaa1038.

(2) https://rcsbsearchapi.readthedocs.io.

(3) Cock, P. J. A.; Antao, T.; Chang, J. T.; Chapman, B. A.; Cox, C. J.; Dalke, A.; Friedberg, I.; Hamelryck, T.; Kauff, F.; Wilczynski, B.; de Hoon, M. J. L. Biopython: freely available Python tools for computational molecular biology and bioinformatics. *Bioinformatics* **2009**, *25*, 1422–1423, DOI: 10.1093/bioinformatics/btp163.

(4) Dana, J. M.; Gutmanas, A.; Tyagi, N.; Qi, G.; O'Donovan, C.; Martin, M.; Velankar, S. SIFTS: updated Structure Integration with Function, Taxonomy and Sequences resource allows 40-fold increase in coverage of structure-based annotations for proteins. *Nucleic Acids Res.* **2019**, *47*, D482–D489, DOI: 10.1093/nar/gky1114.

(5) Rodrigues, J. P. G. L. M.; Teixeira, J. M. C.; Trellet, M.; Bonvin, A. M. J. J. pdb-tools: a swiss army knife for molecular structures. 2018; https://f1000research.com/articles/7-1961.

(6) Amadei, A.; Linssen, A. B. M.; Berendsen, H. J. C. Essential dynamics of proteins. *Proteins: Struct., Funct., Bioinf.* **1993**, *17*, 412–425, DOI: https://doi.org/10.1002/prot.340170408.

(7) Modenutti, C. P. et al. Clamping, bending, and twisting inter-domain motions in the misfold-recognizing portion of UDP-glucose: Glycoprotein glucosyltransferase. *Structure* **2021**, *29*, 357–370.e9, DOI: https://doi.org/10.1016/j.str.2020.11.017.

(8) Zhang, S.; Krieger, J. M.; Zhang, Y.; Kaya, C.; Kaynak, B.; Mikulska-Ruminska, K.; Doruker, P.; Li, H.; Bahar, I. ProDy 2.0: increased scale and scope after 10 years of protein dynamics modelling with Python. *Bioinformatics* **2021**, *37*, 3657–3659, DOI: 10.1093/bioinformatics/btab187.

(9) Göbel, U.; Sander, C.; Schneider, R.; Valencia, A. Correlated mutations and residue contacts in proteins. *Proteins: Struct., Funct., Bioinf.* **1994**, *18*, 309–317, DOI: https://doi.org/10.1002/prot.340180402.

(10) Lapedes, A. S.; Bertrand, G. G.; LonChang, L.; Stormo, G. D. Correlated Mutations in Models of Protein Sequences: Phylogenetic and Structural Effects. *Lect. Notes - Monogr. Ser.* **1999**, *33*, 236–256.

(11) Weigt, M.; White, R. A.; Szurmant, H.; Hoch, J. A.; Hwa, T. Identification of direct residue contacts in protein–protein interaction by message passing. *Proc. Natl. Acad. Sci. U.S.A.* **2009**, *106*, 67–72, DOI: doi:10.1073/pnas.0805923106.

(12) Rao, R. M.; Liu, J.; Verkuil, R.; Meier, J.; Canny, J.; Abbeel, P.; Sercu, T.; Rives, A. MSA Transformer. 2021; https://proceedings.mlr.press/v139/rao21a.html.

(13) Sievers, F.; Higgins, D. G. Clustal Omega for making accurate alignments of many protein sequences. *Protein Sci.* **2018**, *27*, 135–145, DOI: https://doi.org/10.1002/pro.3290.

(14) van Linden, O. P. J.; Kooistra, A. J.; Leurs, R.; de Esch, I. J. P.; de Graaf, C. KLIFS: A Knowledge-Based Structural Database To Navigate Kinase–Ligand Interaction Space. *J. Med. Chem.* **2014**, *57*, 249–277, DOI: 10.1021/jm400378w.

(15) Kanev, G. K.; de Graaf, C.; Westerman, B. A.; de Esch, I. J. P.; Kooistra, A. J. KLIFS: an overhaul after the first 5 years of supporting kinase research. *Nucleic Acids Res.* **2020**, *49*, D562–D569, DOI: 10.1093/nar/gkaa895.

(16) Wang, R.; Zhao, R.; Ribando-Gros, E.; Chen, J.; Tong, Y.; Wei, G.-W. HERMES: Persistent spectral graph software. *Found. Data Sci.* **2021**, *3*, 67–97, DOI: 10.3934/fods.2021006.

(17) Pettersen, E. F.; Goddard, T. D.; Huang, C. C.; Couch, G. S.; Greenblatt, D. M.; Meng, E. C.; Ferrin, T. E. UCSF Chimera–a visualization system for exploratory research and analysis. *J. Comput. Chem* **2004**, *25*, 1605–1612, DOI: 10.1002/jcc.20084.

(18) Šali, A.; Blundell, T. L. Comparative Protein Modelling by Satisfaction of Spatial Restraints. *J. Mol. Biol.* **1993**, *234*, 779–815, DOI: https://doi.org/10.1006/jmbi.1993.1626.

(19) He, X.; Man, V. H.; Yang, W.; Lee, T.-S.; Wang, J. A fast and high-quality charge model for the next generation general AMBER force field. *J. Chem. Phys.* **2020**, *153*, DOI: 10.1063/5.0019056.

(20) Case, D. et al. Amber 2023. 2023.

(21) Maier, J. A.; Martinez, C.; Kasavajhala, K.; Wickstrom, L.; Hauser, K. E.; Simmerling, C. ff14SB: Improving the Accuracy of Protein Side Chain and Backbone Parameters from ff99SB. *J. Chem. Theory Comput.* **2015**, *11*, 3696–3713, DOI: 10.1021/acs.jctc.5b00255.

(22) Jorgensen, W. L.; Chandrasekhar, J.; Madura, J. D.; Impey, R. W.; Klein, M. L. Comparison of simple potential functions for simulating liquid water. *J. Chem. Phys.* **1983**, *79*, 926–935, DOI: 10.1063/1.445869.

(23) Ryckaert, J.-P.; Ciccotti, G.; Berendsen, H. J. C. Numerical integration of the cartesian equations of motion of a system with constraints: molecular dynamics of n-alkanes. *Journal of Computational Physics* **1977**, *23*, 327–341, DOI: https://doi.org/10.1016/0021-9991(77)90098-5.

(24) Darden, T.; York, D.; Pedersen, L. Particle mesh Ewald: An Nlog(N) method for Ewald sums in large systems. *J. Chem. Phys.* **1993**, *98*, 10089–10092, DOI: 10.1063/1.464397.

(25) Roe, D. R.; Cheatham, T. E. I. PTRAJ and CPPTRAJ: Software for Processing and Analysis of Molecular Dynamics Trajectory Data. *J. Chem. Theory Comput.* **2013**, *9*, 3084–3095, DOI: 10.1021/ct400341p.

(26) Morris, G. M.; Huey, R.; Lindstrom, W.; Sanner, M. F.; Belew, R. K.; Goodsell, D. S.; Olson, A. J. AutoDock4 and AutoDockTools4: Automated docking with selective receptor flexibility. *J. Comput. Chem* **2009**, *30*, 2785–2791, DOI: 10.1002/jcc.21256.

(27) Morris, G. M.; Goodsell, D. S.; Pique, M. E.; Lindstrom, W.; Huey, R.; Forli, S.; Hart, W. E.; Halliday, S.; Belew, R.; Olson, A. J. *User Guide: AutoDock Version 4.2*; 2014.

(28) Rooklin, D.; Wang, C.; Katigbak, J.; Arora, P. S.; Zhang, Y. AlphaSpace: Fragment-Centric Topographical Mapping To Target Protein–Protein Interaction Interfaces. *J. Chem. Inf. Model.* **2015**, *55*, 1585–1599, DOI: 10.1021/acs.jcim.5b00103.

(29) Katigbak, J.; Li, H.; Rooklin, D.; Zhang, Y. AlphaSpace 2.0: Representing Concave Biomolecular Surfaces Using β-Clusters. *J. Chem. Inf. Model.* **2020**, *60*, 1494–1508, DOI: 10.1021/acs.jcim.9b00652.

(30) Mariani, V.; Biasini, M.; Barbato, A.; Schwede, T. lDDT: a local superposition-free score for comparing protein structures and models using distance difference tests. *Bioinformatics* **2013**, *29*, 2722–2728, DOI: 10.1093/bioinformatics/btt473.

(31) Hagberg, A. A.; Schult, D. A.; Swart, P. J. Exploring Network Structure, Dynamics, and Function using NetworkX. **2008**, 5.

(32) Möbitz, H. The ABC of protein kinase conformations. *Biochim. Biophys. Acta - Proteins Proteom.* **2015**, *1854*, 1555–1566, DOI: 10.1016/j.bbapap.2015.03.009.

(33) Ung, P. M.-U.; Rahman, R.; Schlessinger, A. Redefining the Protein Kinase Conformational Space with Machine Learning. *Cell Chem. Biol.* **2018**, *25*, 916–924.e2, DOI: 10.1016/j.chembiol.2018.05.002.

(34) Modi, V.; Dunbrack, R. L. Defining a new nomenclature for the structures of active and inactive kinases. *Proc. Natl. Acad. Sci. U.S.A.* **2019**, *116*, 6818–6827, DOI: 10.1073/pnas.1814279116.

(35) Modi, V.; Dunbrack, R. L., Jr Kincore: a web resource for structural classification of protein kinases and their inhibitors. *Nucleic Acids Res.* **2022**, *50*, D654–D664, DOI: 10.1093/nar/gkab920.

(36) Laskowski, R. A.; Swindells, M. B. LigPlot+: Multiple Ligand–Protein Interaction Diagrams for Drug Discovery. *J. Chem. Inf. Model.* **2011**, *51*, 2778–2786, DOI: 10.1021/ci200227u.

(37) Betzi, S.; Alam, R.; Martin, M.; Lubbers, D. J.; Han, H.; Jakkaraj, S. R.; Georg, G. I.; Schönbrunn, E. Discovery of a Potential Allosteric Ligand Binding Site in CDK2. *ACS Chem. Biol.* **2011**, *6*, 492–501, DOI: 10.1021/cb100410m.

(38) Liu, Z.; Su, M.; Han, L.; Liu, J.; Yang, Q.; Li, Y.; Wang, R. Forging the Basis for Developing Protein–Ligand Interaction Scoring Functions. *Acc. Chem. Res* **2017**, *50*, 302–309, DOI: 10.1021/acs.accounts.6b00491.

(39) Hu, L.; Benson, M. L.; Smith, R. D.; Lerner, M. G.; Carlson, H. A. Binding MOAD (Mother Of All Databases). *Proteins: Struct., Funct., Bioinf.* **2005**, *60*, 333–340, DOI: 10.1002/prot.20512.

(40) Wagle, S.; Smith, R. D.; Dominic, A. J.; DasGupta, D.; Tripathi, S. K.; Carlson, H. A. Sunsetting Binding MOAD with its last data update and the addition of 3D-ligand polypharmacology tools. *Sci. Rep.* **2023**, *13*, 3008, DOI: 10.1038/s41598-023-29996-w.
